# Cellular Computational Logic Using Toehold Switches

**DOI:** 10.3390/ijms23084265

**Published:** 2022-04-12

**Authors:** Seungdo Choi, Geonhu Lee, Jongmin Kim

**Affiliations:** Department of Life Sciences, Pohang University of Science and Technology, 77 Cheongam-ro, Pohang 37673, Gyeongbuk, Korea; choisd@postech.ac.kr (S.C.); kunhu0213@postech.ac.kr (G.L.)

**Keywords:** toehold switch, arithmetic operation, RNA–RNA interaction, molecular computing, reversible computing

## Abstract

The development of computational logic that carries programmable and predictable features is one of the key requirements for next-generation synthetic biological devices. Despite considerable progress, the construction of synthetic biological arithmetic logic units presents numerous challenges. In this paper, utilizing the unique advantages of RNA molecules in building complex logic circuits in the cellular environment, we demonstrate the RNA-only bitwise logical operation of XOR gates and basic arithmetic operations, including a half adder, a half subtractor, and a Feynman gate, in *Escherichia coli*. Specifically, de-novo-designed riboregulators, known as toehold switches, were concatenated to enhance the functionality of an OR gate, and a previously utilized antisense RNA strategy was further optimized to construct orthogonal NIMPLY gates. These optimized synthetic logic gates were able to be seamlessly integrated to achieve final arithmetic operations on small molecule inputs in cells. Toehold-switch-based ribocomputing devices may provide a fundamental basis for synthetic RNA-based arithmetic logic units or higher-order systems in cells.

## 1. Introduction

Synthetic biology aims to create technologies for designing and building biological systems with programmable and predictable dynamics [1]. Since the demonstration of synthetic biological circuits in living cells over two decades ago [2,3], considerable progress has been made towards more sophisticated artificial cellular functions, such as feedback oscillation [4], combinatorial logic computation [5,6,7], and information storage [8,9]. In principle, synthetic circuits can be constructed using any biological molecule as a backbone. Natural and engineered protein regulators can provide the framework to implement logic circuits and computing devices [10,11], including de-novo-designed components [12]. Still, the construction of synthetic biological circuits presents numerous challenges, including the lack of composability [13], limited modularity [14], unpredictable cross-reactivity [15], cellular resource usage [16,17], and unexpected idiosyncratic behavior in real-world applications [18]. Therefore, novel approaches for synthetic biological circuits, including the development of readily characterized, standardized, and modular components are required to overcome the innate difficulties in managing and programming cellular behavior towards large, complex synthetic systems [19].

Nucleic-acid-based genetic devices have made remarkable progress in molecular computing and may provide the required platform for scalable synthetic biological systems. Complex logic circuits and advanced computing systems have been implemented using toehold-mediated strand displacement, including a bistable circuit [20], a square-root circuit [21], neural networks for memory [22] and pattern recognition [23], and an arithmetic logic unit [24]. Furthermore, these molecular computing systems are amenable to computational design and analysis [25,26]. While DNA strand-displacement circuits have been demonstrated in live cells [27] and for live-cell imaging [28], DNA logic gates are generally not suitable for in vivo applications due to the challenges in generating single-stranded DNAs [29] and maintaining stable gate configurations [30] in cells. In comparison, RNA provides several advantageous characteristics for synthetic biological computing devices. RNA strands can be easily programmed to interact in a designed fashion due to the single-stranded nature of RNA. The co-transcriptional folding of RNA allows the formation of stable secondary structures [31], suitable for natural and synthetic riboswitches [32]. Furthermore, RNA signals can be easily modulated in a tunable manner [33] and can also be amplified with several RNA and protein counterparts [34,35,36,37,38]. Therefore, RNA has been exploited as a platform to engineer gene expression programs that operate robustly in vivo [39,40,41,42,43,44].

**Figure 1:**
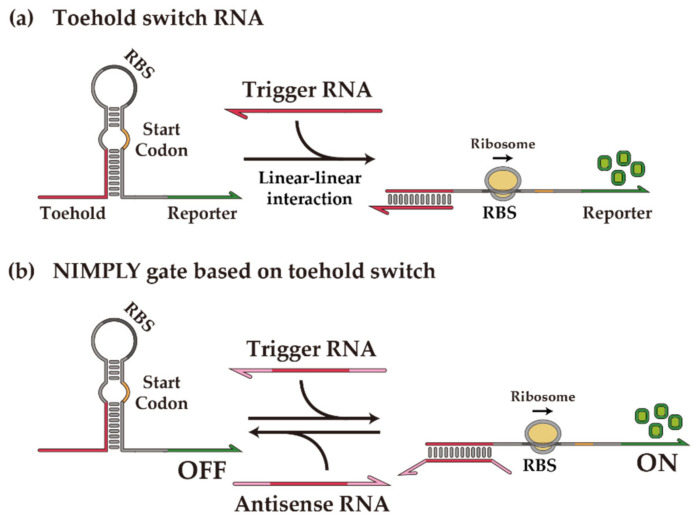
De-novo-designed toehold switch and toehold-switch-based NIMPLY gate. (**a**) Scheme of toehold switch operation. The toehold switch has repressed the translation state through the secondary structure sequestering the RBS and start codon. Linear-linear interaction between the toehold switch and the trigger RNA exposes the RBS and start codon with the strand displacement process; therefore, the translation of the downstream gene is resumed. (**b**) Scheme of NIMPLY gate operation. Antisense RNA has extended overhang sequences at both ends and can inhibit the trigger RNA through sequence displacement or complementary binding. Thus, the toehold switch is reverted to the initial OFF state.

Building on the success of synthetic RNA regulatory parts and inspired by natural RNA regulators [45], several de-novo-designed RNA regulators have been utilized for synthetic biological devices with a large library of well-characterized parts [40,41]. As an example, toehold switches control gene expression in trans via well-established Watson-Crick base pairing of switch and trigger RNA molecules (Figure 1a) [40]. Unlike conventional riboregulators [39], the toehold switches remove nearly all the sequence constraints, exhibit a wide dynamic range, and show excellent programmability with a large library of orthogonal parts. The versatility of toehold switches for synthetic genetic circuit construction is exemplified by the recent developments in cellular logic computation [46], translational repressing riboregulators [47], incoherent feed-forward loop circuits [48], synthetic transcription terminators [49], the protein quality control system [50], modulators of riboswitch circuits [51], and regulators of mammalian cells [52]. Beyond cellular circuits, the toehold switches find use in other platforms, such as cell-free systems [53,54,55,56,57] and paper-based diagnostic devices [58,59,60] for broader applications.

In particular, toehold switches may form the basis for constructing an arithmetic logic unit (ALU) in vivo. A generalized toehold switch architecture, termed ribocomputing devices, concatenated several toehold switch sensor domains and utilized the self-assembly of RNA species to compute multi-input AND/OR/NOT operations [46]. The design flexibility of toehold switches, if effectively utilized, can lead to the streamlined design and construction of a basic form of synthetic biological ALU. Previous work has demonstrated basic ALUs, including a half adder and a half subtractor, in bacteria and mammalian cells [5,61,62]. These binary calculators can perform bitwise calculations across two 1-bit input signals and serve as building blocks for higher-level systems. A half adder takes two bits of information and generates two output bits: one for the sum and one for the carry. The sum bit can be calculated via an XOR gate, and the carry bit can be calculated via an AND gate. Thus, it is straightforward to calculate the carry bit, as previously shown, but a toehold-switch-based XOR gate needs to be engineered. Unlike previous XOR gate implementation, for example, in vitro [63], in prokaryotes [61,62,64,65], and in eukaryotes [66,67,68], a toehold-switch-based XOR gate is an RNA-only synthetic logic device. Building on previous work in which an antisense RNA that titrates a cognate trigger RNA molecule can be used for a NIMPLY gate operation (Figure 1b), a toehold-switch-based XOR gate with a compact architecture can be obtained by concatenating two orthogonal switches in an OR-gate fashion. A half subtractor can be analogously constructed with an XOR gate and a NIMPLY gate. In addition, a Feynman gate, one of the reversible logic gates that map input and output signals in a one-to-one manner [69], can be obtained using an XOR gate and a BUFFER gate. In summary, we present the binary operation of an XOR gate, cellular arithmetic operations with a half adder and a half subtractor, and a Feynman gate in *E. coli* using a de-novo-designed toehold switch and antisense RNAs. Synthetic RNA-based ALUs could lay the foundation for making sophisticated molecular devices with neural-network-like capabilities for biomedical applications.

## 2. Results

### 2.1. XOR Gate

#### 2.1.1. Design of XOR Gate with Toehold Switches

The NIMPLY gate often used in synthetic biology and genetic circuits [61,70] was previously demonstrated using toehold switches [46]. A NIMPLY B is equivalent to A AND (NOT B), and an XOR gate can be constructed using two NIMPLY gates connected via an OR gate as follows: A XOR B = (A NIMPLY B) OR (B NIMPLY A). Thus, we sought to first demonstrate two orthogonal NIMPLY gates. The mechanism for NIMPLY gates is analogous to previous work where the switch RNA is activated by the trigger RNA (A), and the antisense RNA (B) deactivates the trigger RNA via direct hybridization or toehold-mediated strand displacement to separate the trigger RNA bound to the switch RNA. The extended overhang sequences at both ends provide the thermodynamic driving force to shift the equilibrium towards trigger and antisense RNA binding rather than trigger and switch RNA binding.

**Figure 2:**
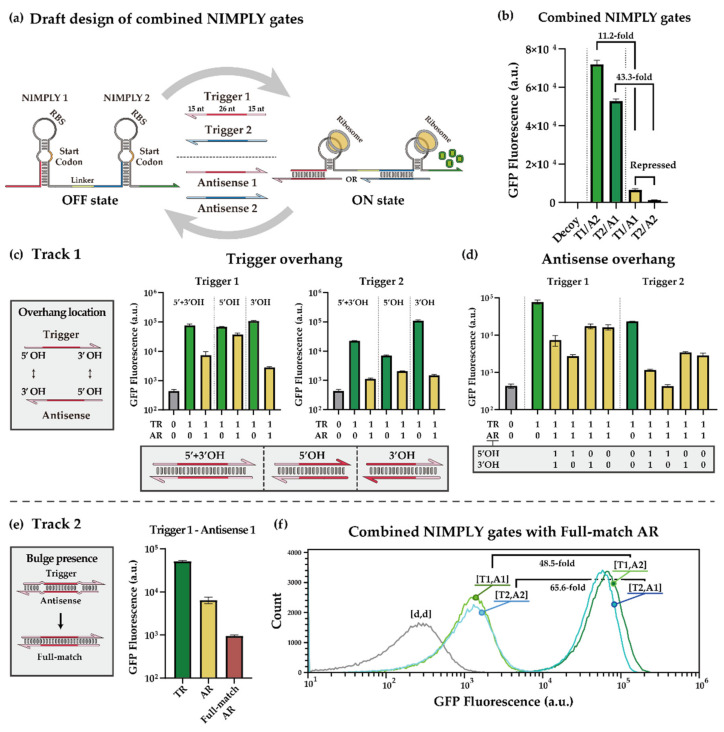
Design of combined NIMPLY gates and optimization strategies. (**a**) Scheme of combined NIMPLY gates composed of two orthogonal NIMPLY gates connected by a 9-nt linker. Trigger and antisense RNAs control the translation states of NIMPLY gates. (**b**) Performance of initial design for combined NIMPLY gates. T1 and T2 denote trigger RNAs that activate NIMPLY gates 1 and 2, respectively, and A1 and A2 denote antisense RNAs that annihilate trigger RNAs 1 and 2, respectively. (**c**,**d**) Effect of the location of extended overhangs on trigger and antisense RNAs. TR and AR indicate trigger RNA and antisense RNA, respectively. Absence of trigger (TR = 0) indicates that only the NIMPLY gate RNA is present. (**e**) Effect of the presence of bulge on antisense RNA. TR and AR indicate trigger RNA and antisense RNA, respectively. Full match means that no bulge was introduced in the antisense RNA design. (**f**) Flow cytometry GFP fluorescence histograms for the NIMPLY complex with full-match antisense RNAs. T1, T2, A1, and A2 indicate trigger and antisense RNAs for switches 1 and 2 as above, and d represents a decoy RNA that does not interact with the switch RNA. T7 RNA polymerase was induced by 1 mM IPTG in *E. coli* BL21 DE3 strain. GFP fluorescence was measured on the flow cytometry (error bars indicate ± SD from three biological replicates). Cellular autofluorescence was subtracted in all cases. Autofluorescence level was measured from cells not bearing a GFP-expressing plasmid.

To implement a NIMPLY gate in *E. coli*, the three circuit components—switch RNA, trigger RNA, and antisense RNA—should be selected from the existing library with appropriate modifications (Figure 2a). We selected a pair of second-generation toehold switches with large dynamic range and strong orthogonality. These two switches are connected with a 9-nt linker sequence to create an OR gate, as previously demonstrated [46]. The overhang sequences of both trigger RNAs and antisense RNAs were designed via the RNA secondary structure prediction software NUPACK [71,72,73,74,75] (Table S1). Fifteen nucleotide overhangs were attached to both ends of the trigger and antisense RNAs, and a single nucleotide bulge was inserted between the overhang region and the switch binding domain to prevent the formation of long double-stranded RNA that could be targeted for degradation by RNase III [76,77]. The design candidates were analyzed for ensemble defect [78], overhang accessibility, and crosstalk in silico to select the best designs to be tested in experiments.

The NIMPLY gates were tested in *E. coli* BL21 DE3 strain with the switch, trigger, and antisense RNAs expressed from separate low, medium, and high copy plasmids, respectively. The RNA components were under the control of a T7 promoter, and genomically encoded T7 RNA polymerase was induced by Isopropyl β-d-1-thiogalactopyranoside (IPTG). GFP was used to characterize the switch output performance via flow cytometry. First, the OR gate functionality was verified in the absence of antisense RNAs, where the GFP fluorescence was increased at least 100-fold in the presence of either trigger RNAs (Figure S1). Next, the switch, trigger, and antisense RNAs were expressed together in cells to evaluate the function of the NIMPLY gates. The design with the least expected intramolecular and intermolecular structures showed the best performance among the candidates (Figure 2b). The performance was evaluated by dividing the ON state, with a cognate trigger RNA and a non-cognate antisense RNA, by the repressed state, with cognate trigger and antisense RNA pairs. Consequently, we observed increases of 11.2-fold and 43.3-fold for T1-A1 and T2-A2, respectively. Other design candidates with expected secondary structures within trigger RNAs showed relatively poor functionality (Figure S2). Since the design variants on previous NIMPLY gate designs [46] were not extensively characterized, we aimed to further explore and optimize the design choices to enhance the functionality of the NIMPLY gates and hence the performance of the synthetic XOR gate.

#### 2.1.2. Optimization Strategies for Toehold-Switch-Based XOR Gate

For the design variants of the trigger and antisense RNAs within the NIMPLY gate, we mainly adjusted the location of the extended overhangs and the presence of bulge. First, we investigated whether the location of the overhang could affect the functionality of the trigger or antisense RNAs. The GFP fluorescence output for trigger RNA 2 with overhangs showed a stronger reduction than the trigger RNA 2 without the overhang sequences (Figure S3). We hypothesized that the close proximity of RBS within switch 2 and the 5′ overhang of trigger RNA 2 affect the access of RBS through steric hindrance. Therefore, trigger RNA variants with only a 5′ overhang or a 3′ overhang were constructed, and the impact of the overhang location on the switch performance was investigated. Trigger RNAs with only a 5′ extended overhang showed weak repression by antisense RNA for switch 1 and weak activation for switch 2, indicating that the 5′ extended overhang could reduce performance. On the other hand, trigger RNAs with only a 3′ extended overhang showed improved performance for both switches compared to the trigger RNAs with both 5′ and 3′ overhang domains (Figure 2c). The antisense RNAs were similarly modified to test the impact of overhang domains: antisense RNAs with only a 5’ overhang showed improved fold repression, while antisense RNAs with only a 3’ overhang showed weak repression comparable to the antisense RNAs without the extended overhangs (Figure 2d).

Other design candidates were analyzed for the impact of overhang locations on the trigger and antisense RNAs, and a similar trend was observed (Figure S4). Although the 5′ extended overhang can be considered disposable, simply removing the existing 5′ overhang caused crosstalk in some cases because it was not considered during the design phase (Figure S5). Fortunately, the apparent crosstalk was negligible when the 3’ overhang trigger RNAs were paired with antisense RNAs with both overhang sequences (Figure S6). Additionally, an expanded hairpin loop of the switch RNA was explored to help reduce the potential steric hindrance of trigger RNA on the RBS. The increased hairpin size in switch RNA increased the ON level expression but also generally increased the OFF-state leakage (Figure S7). Together, we observed the impact of extended overhang locations on both trigger and antisense RNAs and trade-offs in the switch RNA hairpin loop size on the performance of NIMPLY gates.

To further enhance the functionality of the NIMPLY gates, we investigated the effect of bulges within the trigger–antisense RNA complex on the repression efficiency. The antisense RNA presumably works in one of two ways: (1) dissociating the trigger from the switch or (2) capturing the trigger freely floating in the cytoplasm [46]. Single nucleotide bulges located between the overhang and switch binding domain can act as an energy barrier to the strand displacement pathway that removes the trigger from the switch [79,80]; in that case, the direct hybridization mechanism would be predominant. In order to increase the repression efficiency of antisense RNA, an antisense RNA with extended overhangs but without bulges was designed and tested. The repression efficiency was enhanced nearly 10-fold on trigger 1 (Figure 2e), and the combined NIMPLY gates with optimization exhibited 48.5-fold and 65.6-fold improvements, respectively (Figure 2f). Therefore, we successfully constructed two orthogonal NIMPLY gates with large dynamic ranges using optimization strategies on switch, trigger, and antisense RNA designs. These may serve as useful strategies for other toehold-switch-based logic circuit designs and potentially for other RNA regulatory devices as well.

### 2.2. In Vivo Characterization of XOR Gate

**Figure 3:**
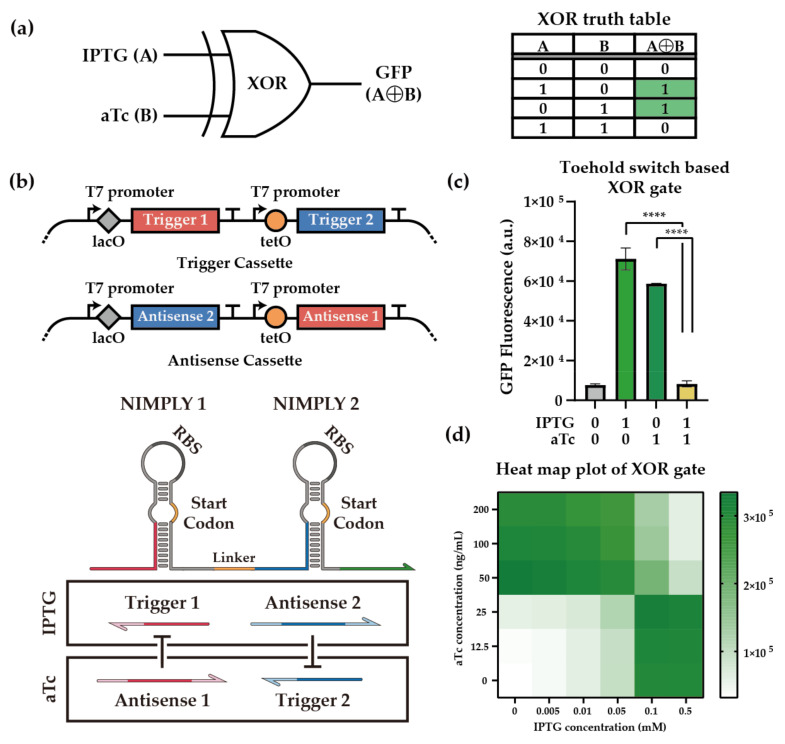
Toehold-switch-based XOR gate. (**a**) Schematics of XOR gate configuration. IPTG and aTc were used as input signals A and B for the XOR gate, and the output signal was visualized through GFP fluorescence. The truth table of the XOR gate indicated the ON and OFF states of the XOR gate depending on the combination of inducer molecules. (**b**) Genetic blueprint of trigger and antisense cassettes and schematics of the XOR gate. Lac operator was placed upstream of T1 and A2, and Tet operator was placed in front of T2 and A1. The optimized extended overhangs were used. (**c**) Performance of toehold-switch-based XOR gate. T7 RNA polymerase was induced with the pretreatment of 1% (*w*/*w*) arabinose in *E. coli* BL21 AI strain. XOR gate components were induced by 0.5 mM IPTG and 100 ng/mL aTc. GFP fluorescence was measured via flow cytometry. Cellular autofluorescence was subtracted in all cases. Autofluorescence level was measured from cells not bearing a GFP-expressing plasmid. Statistical analysis was performed to compare each state of the XOR gate. (Two-tailed Student’s *t*-test; **** *p* < 0.0001; Error bars indicate ± SD from three biological replicates) (**d**) Heat map plot of XOR gate output. The color scale was ranged between the minimum and maximum values of the XOR gate output. IPTG and aTc were treated in gradient concentration as described in the table. Each point of the heat map indicates the median value of three replicates.

Encouraged by the optimized NIMPLY gates, we then aimed to construct an XOR gate with two chemical inducers as inputs: IPTG and anhydrotetracycline (aTc). An XOR gate provides a true output with an odd number of true inputs (Figure 3a). In the case of the chemically inducible XOR gate that we aimed to construct, the GFP output should be high when either IPTG or aTc is present, but not both. To achieve this, Lac and Tet operators were strategically placed downstream of T7 promoters that drive the expression of trigger and antisense RNAs, such that the trigger RNA of one NIMPLY gate and the antisense RNA of the other NIMPLY gate are simultaneously induced by the same chemical inducer for both inducers (Figure 3b). Specifically, an IPTG induction promotes the expression of trigger RNA 1 and antisense RNA 2, such that the output GFP expression is high. The process works similarly for aTc induction. However, the simultaneous treatment of both inducers results in the expression of both trigger RNAs as well as both antisense RNAs, such that the GFP expression is inefficient. While the Lac and Tet operator sequences are also expressed upon the expression of the trigger and antisense RNAs, the expected secondary structure changes on the core signaling parts of the trigger and antisense RNAs were not noticeable (Figure S8).

At the molecular level, the optimized NIMPLY gates previously characterized were used along with the overhang deletion and bulge deletion strategies. The switch RNA that combines two orthogonal switches in an OR-gate fashion is expressed from a low copy plasmid. To facilitate strong repression by the antisense RNAs, both the trigger RNAs were expressed from a medium copy plasmid, and both the antisense RNAs were expressed from a high copy plasmid. The performance of the XOR gate was evaluated in *E. coli* BL21 AI strain, where genomically encoded T7 RNA polymerase was induced by arabinose. A number of basic molecular interactions were verified for the XOR gate: the crosstalk between the switch and antisense RNAs was negligible (Figure S9); both trigger RNAs, despite the attached additional operator sequences, could turn on the switch RNAs (Figure S10); the antisense RNAs could annihilate the cognate trigger RNA activities, as expected, with little crosstalk (Figure S11). When all the components were put together and the chemical inducers were used, the XOR gate functioned as expected, with a high ON state for either IPTG or aTc input but with a low OFF state for no inducer or both inducer cases (Figure 3c). An XOR gate using trigger RNAs with both 5′ and 3′ overhangs was also shown to be functional, albeit with a reduced ON state for trigger 2 (Figure S12). Furthermore, the GFP output pattern changed sharply as the concentration of inducers was adjusted, indicating that the XOR gate showed a switch-link function suitable for digital circuits (Figure 3d). When incorporated within larger logic circuits, this digital logic ensures an all-or-none response to a variety of inputs and provides a robust output signal regardless of input perturbations [81], thus conveying information with less noise and high accuracy for decision-making processes [82,83].

#### 2.2.1. Cellular Arithmetic Operation of a Half Adder and a Half Subtractor

**Figure 4:**
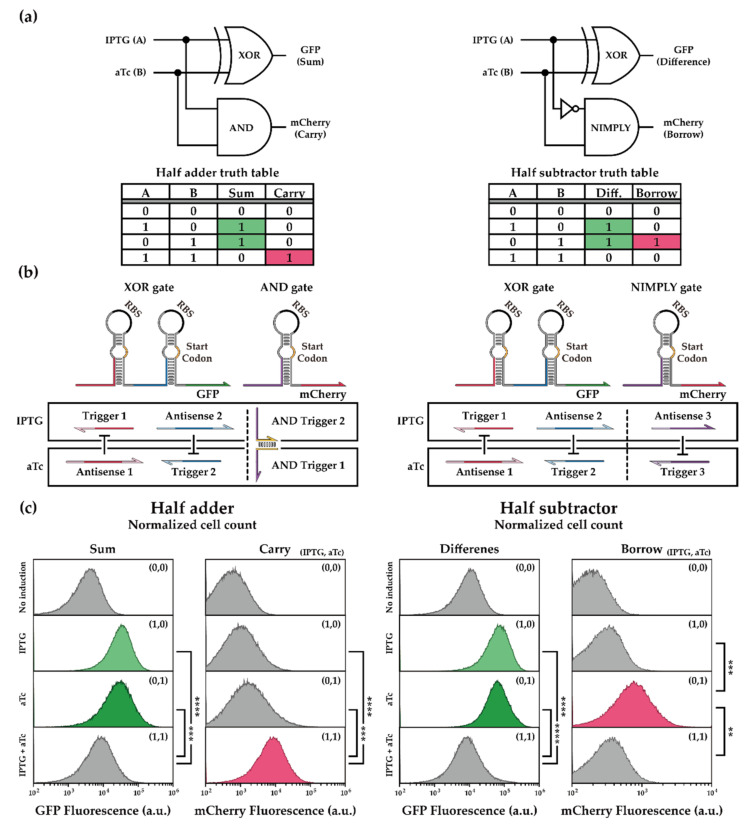
Binary operation of half adder and half subtractor. (**a**) Schematic of half adder and half subtractor configurations. IPTG and aTc were used as input signals, and the output signals were visualized through GFP and mCherry fluorescence. GFP was assigned to the XOR gate output, and mCherry was assigned to AND or NIMPLY gate output. The truth table of the binary calculators indicated the ON and OFF states of each binary calculator depending on the combination of inducer molecules. Diff. denotes the Difference bit of the half subtractor. (**b**) Schematic of toehold-switch-based half adder and half subtractor. Trigger and antisense RNAs under the same inducer control are shown in boxes. (**c**) Flow cytometry GFP and mCherry fluorescence histograms for the half adder and the half subtractor. The presence of IPTG and aTc was displayed within each panel in brackets. T7 RNA polymerase was induced with the pretreatment of 1% (*w*/*w*) arabinose in *E. coli* BL21 AI strain. RNAs of the half adder and the half subtractor were induced by 1 mM IPTG and 200 ng/mL aTc. Statistical analysis was performed for comparing each state of the binary calculators. (Two-tailed Student’s *t*-test; ** *p* < 0.01; *** *p* < 0.001; **** *p* < 0.0001).

Building on the RNA devices that were modularized and rigorously characterized earlier, the logical complexity of synthetic RNA circuits can be further increased. As a test case, we focused on basic binary calculators: the half adder and the half subtractor. A half adder takes two input bits and generates two output bits that require an XOR gate for SUM output and an AND gate for CARRY output (Figure 4a). A half subtractor can be analogously constructed, where an XOR gate computes the DIFFERENCE output and a NIMPLY gate computes the BORROW output. Fortunately, a high-performance AND gate was available from the toehold switch library, and another orthogonal NIMPLY gate was constructed with available toehold switches after NUPACK analysis. The functionality of the AND gate and the NIMPLY gate were verified in isolation (Figure S13). Then, these new genetic elements were incorporated into expression cassettes in the same plasmid backbones as before. The XOR gate with GFP output was used to compute the SUM and DIFFERENCE output bits, and the newly introduced AND gate and NIMPLY gate with mCherry output were used to compute the CARRY and BORROW output bits in the half adder and half subtractor, respectively (Figure 4b). To investigate the performance of the binary calculators at the single-cell level, we characterized the system by flow cytometry. For all input combinations, the half adder and half subtractor showed correct ON and OFF states with statistically significant differences (Figure 4c and Figure S14). Nevertheless, as the genetic complexity and the number of heterologous expression cassettes increased, a concomitant decrease in circuit performance was observed when compared to the single XOR gate. Therefore, we checked all combinations of RNA–RNA interactions with NUPACK 4.0.0.25 and confirmed that on-target MFE structures were maintained, albeit with some unintended crosstalk interactions (Table S2). Further improvements in circuit elements, as well as contexts such as promoter arrangements and spacer sequences, may allow for the successful implementation of even more complex circuits such as a full adder.

#### 2.2.2. Cellular Reversible Logic Operation of Feynman Gate

**Figure 5:**
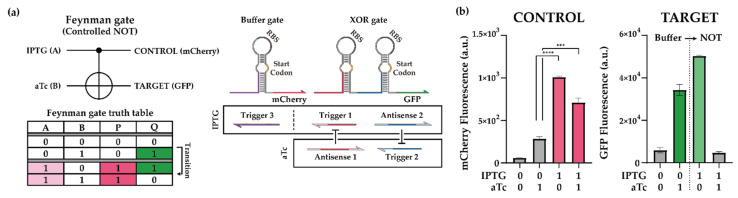
Demonstration of toehold-switch-based Feynman gate. (**a**) Schematic of the Feynman gate. Truth table of the Feynman gate indicated the ON and OFF states for each of the binary calculators depending on the combination of inducer molecules. Transition denotes the functional transition of a Buffer gate to a NOT gate. IPTG and aTc were used as input signals. Trigger and antisense RNAs under the same inducer control are shown in boxes. (**b**) Performance of Feynman gate. T7 RNA polymerase was induced with the pretreatment of 1% (*w*/*w*) arabinose in *E. coli* BL21 AI strain. RNAs of Feynman gate were induced by 1 mM IPTG and 200 ng/mL aTc. GFP and mCherry fluorescence were measured on flow cytometry. Cellular autofluorescence was subtracted in all cases. Autofluorescence level was measured from cells not bearing a GFP- or mCherry-expressing plasmid. Statistical analysis was performed for comparing each state of the Feynman gate. (Two-tailed Student’s *t*-test; *** *p* < 0.001; **** *p* < 0.0001; Error bars indicate ± SD from three biological replicates).

Reversible computing, a nonconventional form of computing with one-to-one mapping of inputs and outputs, may be useful for biomolecular diagnostic and sensing applications. One of the reversible computing devices, a Feynman gate, can also be constructed using a method similar to other binary calculators, using an XOR gate and a BUFFER gate. Due to the unique output patterns, it is also called as a controlled NOT gate when the output signal (Q) changes from BUFFER gate to NOT gate in response to the input signal (Figure 5a). At the molecular level, we executed the same set of sequences as those for the half subtractor, except that a trigger 3 without extended overhangs was used. The RNA-based Feynman gate possessed both a functional switching ability and the capacity for information storage (Figure 5b and Figure S15). The circuit acted as a BUFFER gate for input B in the absence of input A, whereas the circuit functioned as a NOT gate for input B in the presence of input A. Furthermore, information about the input combinations was preserved in the Feynman gate because of the one-to-one manner of input to output mapping. Overall, we demonstrated that toehold-switch-based ribocomputing designs may prove useful for reversible computing in cells.

## 3. Discussion

In this study, we present the binary operation of XOR gates, cellular arithmetic operation including a half adder and a half subtractor, and a Feynman gate in single-cell *E. coli* using de-novo-designed toehold switches and antisense RNAs. A systematic approach was taken where the basic building block, a NIMPLY gate, was optimized and then subsequently used for an XOR gate, which in turn could be used for basic ALUs. While the NIMPLY gate design was previously demonstrated [46], it was simply used as a proxy for a NOT gate, and further optimization of its performance was limited. Hence, we investigated several design candidates using a number of parameters, including the ensemble defect, the overhang domain accessibility, and the cross-reactivity in silico. The design candidates with accessible overhang domains generally showed better performance (Figure 2b and Figure S2). Crucially, several optimization strategies can improve the performance of NIMPLY gate designs. A thorough analysis of the impact of extended overhang sequences revealed that a 3′ extended overhang on the trigger RNA and a 5′ extended overhang on the antisense RNA improved performance compared to having overhangs on the other location or on both sides. We reasoned that the negative effect of a 5′ extended overhang on the trigger RNA might be due to the potential interference on the ribosome binding to the RBS of the switch. One piece of evidence in support of this hypothesis is that the trigger with a 5′ extended overhang showed improved functionality for toehold switches with increased loop length. Although the mechanistic reasoning on the impact of overhang locations on the antisense RNA is unclear, there still may be physical interference during the initiation stage of trigger and antisense RNA interactions. Recognizing that the single nucleotide bulges located between the overhang and switch binding domains can act as an energy barrier to strand displacement [79,80], we tested the antisense RNA with no bulges and observed improved repression efficiency. These optimization strategies laid the foundation for constructing more complex systems building on the NIMPLY gate designs.

Notably, the antisense RNA designs can be extended to other related synthetic RNA regulators. As an example, a recently reported 3-way junction (3WJ) repressor [47] can be analogously regulated using the antisense RNA design for trigger RNAs (Figure S16). The output characteristics can be considered as an implementation of an IMPLY gate (Figure S17). If applied to the previously reported NOR gate constructed using the 3WJ repressor, an XNOR gate could be constructed similar to the toehold-switch-based XOR gate reported here (Figure S17). Recognizing that NAND gate outputs are distinct from those of XOR gates in the no input case, a NAND gate can be constructed from the current XOR gate by changing the inducible promoters of the trigger RNAs to constitutive promoters (Figure S17). Another important class of de-novo-designed RNA regulator, the small transcription activating RNA (STAR) [41,42], was also subject to antisense RNA-based regulation (Figure S18). Both the T181- and AD1-based STAR designs were successfully regulated using antisense RNA that targets the toehold-binding domain and several bases within the stem-binding domain of the STAR trigger RNAs. Together, these findings indicate that the antisense RNA regulators can be adapted in a straightforward manner to other synthetic RNA regulators and can potentially be used to scale up the complexity of synthetic RNA-based regulatory circuits.

The successful demonstration of a synthetic XOR gate can be seen as a benchmark for systematic synthetic gene circuit construction. Previously, several lines of work demonstrated RNA-based XOR gates, including sRNA [62], miRNA [67], and gRNA [68] that encompass bacterial cells as well as mammalian cell lines. Still, the repression mechanism within the XOR gates relied on protein regulators such as phage-encoded λ repressor protein (CI) [62]. Thus, our demonstration of an RNA-only XOR gate provided a distinct design approach for synthetic XOR gates with performance comparable to the previous work in bacterial cells [62]. More importantly, these RNA-only logic gates can be seamlessly combined for basic ALUs, a half adder and a half subtractor, with performance rivaling previous work [61,62]. Despite thorough in silico analysis and screening for optimized system composition, the performance of basic ALUs showed fold changes less than those of individual gates. There are a number of potential pitfalls in the circuit construction, including the leaky expression of promoters, the unexpected interaction between components, and the cellular burden on synthetic RNA expression. Fortunately, these shortcomings can be mitigated with alternative tightly controlled promoters, such as AraBAD or rhaBAD [84,85,86] or novel synthetic promoters [87], and by the division of load to different cell populations with multicellular networks [88,89,90].

Herein, we provided a framework for constructing several synthetic RNA-only basic ALUs with de-novo-designed toehold switches at the single-cell level. This design paradigm offers excellent programmability with simple structural specifications rather than sequence constraints. First, the concatenation of switch RNAs can effectively reduce the encoding space of genetic programs and, therefore, enable the operation of complex systems in *E. coli*. Second, the ALUs can be designed with almost no sequence constraints with in silico screening and optimization. Third, a large library of orthogonal toehold switches provides the required parts for building complex systems. Lastly, the system inherits the general advantages of RNA-based operations, including a fast response time, reduced resource usage, and multiplexing [46,91,92]. Recent developments on degradation-tunable RNAs in combination with toehold switches may provide further design flexibility [93]. Notably, a variety of ALU circuits using DNA strand displacement reactions [24] showcases the power of nucleic-acid-based molecular computations. Still, the demonstrations of ALUs in living cells are limited in complexity and scope. The toehold-switch-based ribocomputing circuits could open a new avenue to exploring the rich design space of synthetic RNA-based ALUs, building up to higher-order systems such as a full adder and a full subtractor, ultimately leading to neural-network-like functions in cells.

## 4. Materials and Methods

### 4.1. E. coli Strains and Plasmid Construction

The following *E. coli* strains were used in this study: BL21 DE3 (Invitrogen; F^−^ *omp*T *hsd*S_B_ (r_B_^−^ m_B_^−^) *gal dcm),* BL21 AI (Invitrogen; F^−^ *omp*T *hsd*S_B_ (r_B_^−^ m_B_^−^) *gal dcm ara*B::T7RNAP-*tet*A), and DH5α (Invitrogen; *endA1 recA1 gyrA96 thi-1 glnV44 relA1 hsdR17*(r_K_^−^ m_K_^+^) λ^−^).

The backbones for the plasmids used in this research were taken from the commercial vectors pET15b, pCDFDuet, pCOLADuet, and pACYCDuet (EMD Millipore). The switch RNA of the NIMPLY complex was constructed using ACTS Type II N3 and ACTS Type II N7 from previous research [46] and was constructed in pACYCDuet. All the trigger RNAs and trigger cassettes were constructed in pCDFDuet. All the antisense RNAs and antisense cassettes were constructed in pET15b. The switch RNAs of the AND gate and the NIMPLY gate were constructed in pCOLADuet. All constructs were cloned via blunt end ligation [94], Gibson Assembly [95], circular polymerase extension cloning (CPEC) [96], and/or round-the-horn site-directed mutagenesis [97]. The plasmid architecture and specific part sequences are listed in Tables S3–S11. Plasmids were constructed in *E. coli* DH5α and purified using the EZ-PureTM plasmid Prep Kit. Ver. 2 (Enzynomics). Plasmid sequences were confirmed by DNA sequencing after every cloning step. Plasmids were transformed through chemical transformation [98].

### 4.2. Cell Culture and Induction Condition

For in vivo experiments, *E. coli* BL21 DE3 and AI strains were used; they contain chromosomally integrated T7 RNA polymerase under the control of IPTG-inducible lacUV5 promoter and arabinose-inducible P_BAD_ promoter, respectively. For the in vivo characterization of the NIMPLY complex in Figure 2, chemically transformed *E. coli* BL21 DE3 cells were cultured on LB agar plates (1.5% agar) with appropriate antibiotics: pACYCDuet (25 μg/mL Chloramphenicol), pCDFDuet (50 μg/mL Spectinomycin), and pET15b (100 μg/mL Ampicillin). Single colonies were grown overnight (~16 h) in 96-well plates with shaking at 800 rpm, 37 °C. Overnight cultures were diluted 1/100-fold into fresh media and returned to shaking (800 rpm, 37 °C). After 80 min, cell cultures were induced with 1 mM IPTG (Promega) and returned to the shaker (800 rpm, 37 °C) until fluorescence measurement after 3 h 30 min. For the experiments on the toehold-switch-based XOR gates, a half adder, a half subtractor, and a Feynman gate, chemically transformed *E. coli* BL21 AI cells were cultured on LB agar plates (BD biosciences; 1.5% agar) with appropriate antibiotics. All antibiotics were purchased from Gold biotechnology: pACYCDuet (25 μg/mL Chloramphenicol), pCOLADuet (50 μg/mL Kanamycin), pCDFDuet (50 μg/mL Spectinomycin), and pET15b (100 μg/mL Ampicillin). Single colonies were grown overnight (~16 h) in 96-well plates with shaking at 800 rpm, 37 °C. Overnight cultures were diluted 1/100-fold into fresh media and returned to shaking (800 rpm, 37 °C). After 80 min, cell cultures were induced with 1% (*w*/*w*) arabinose (Gold biotechnology) to produce T7 RNA polymerase for 1 h with shaking (800 rpm, 37 °C). Then the cell cultures were induced with 0.5 mM IPTG, 100 ng/mL aTc (Takara) for the XOR gates and 1 mM IPTG, 200 ng/mL aTc for a half adder, a half subtractor, and a Feynman gate, and returned to the shaker (800 rpm, 37 °C) until fluorescence measurement after 3 h 30 min.

### 4.3. Microplate Reader Analysis

For the experiment on the XOR gate with a gradient concentration of chemical inducers (Figure 3d), 200 µL of cell cultures were added per well on a 96-well Black Plate 33,396 (SPL) after 1 mM IPTG induction. GFP fluorescence (excitation: 479 nm, emission: 520 nm), mCherry fluorescence (excitation: 587 nm, emission: 610 nm), and OD600 were measured in a Synergy H1 microplate reader (Biotek) running Gen5 3.08 software. GFP and mCherry fluorescence levels were normalized as follows: the fluorescence of LB blank was subtracted for background normalization, and a measured fluorescence value was divided by its OD600. The number of biological replicates was three for in vivo experiments.

### 4.4. Fluorescence Measurements Using Flow Cytometry

GFP fluorescence was measured using flow cytometry (CytoFLEX S, Beckman Coulter, Brea, CA, USA) after fixation. The cell pellet was resuspended with 2% (*w/v*) para-formaldehyde solution (Sigma Aldrich) and fixed for 15 min at room temperature. After fixation, samples were washed twice using 1X phosphate-buffered saline (PBS; Enzynomics). Fixed cells were diluted by a factor of ~5 into 1X PBS. Cells were detected using a forward scatter (FSC) trigger, and at least 100,000 events were recorded for each measurement. Cell population was gated according to the FSC and side scatter (SSC) distributions as described previously [40]. To evaluate the circuit output, the fluorescence of GFPmut3b-ASV was measured on a FITC channel, excited with a 488-nm and detected with a 525/40-nm bandpass filter. The fluorescence of mCherry was measured on ECD/mCherry channel, excited with a 561-nm and detected with a 610/20-nm bandpass filter. GFP and mCherry fluorescence histograms yielded unimodal population distributions, and the geometric mean was employed for the average fluorescence across the approximately log-normal fluorescence distribution from three biological replicates. The fold repression of GFP and mCherry were then calculated by taking the average fluorescence from the cognate RNA-expressing case and dividing it by the fluorescence from the antisense RNA-expressing case. Cellular autofluorescence was subtracted in all cases.

## 5. Conclusions

Expanding the pool of programmable and predictable logic gates is one of the important goals of synthetic biology. Here, we aimed to demonstrate several RNA-only logic gates using toehold switches and antisense RNAs. RNA-only XOR gates, serving as the basic building blocks of arithmetic logic circuits, were constructed using orthogonal NIMPLY gates. Subsequently, the optimized synthetic logic gates were incorporated into arithmetic operations and reversible logic gates via a bottom-up approach in single-cell *E. coli*. In conclusion, toehold-switch-based ribocomputing devices can provide a platform for synthetic RNA-based higher-order circuits in cells.

## Data Availability

The data presented in this study are available on request from the corresponding author.

## Supplementary Materials

The following are available online at https://www.mdpi.com/article/10.3390/ijms23084265/s1.
