# Supplementary material for: Cellular Computational Logic Using Toehold Switches
Source: Int J Mol Sci. 2022 Apr 12;23(8):4265. doi: 10.3390/ijms23084265 (PMC9033136; doi:10.3390/ijms23084265)
Supplement: Supplementary file 1 [file ijms-23-04265-s001.zip › ijms-1649879-supplementary.pdf]

International Journal of *Molecular Sciences*

Article

Cellular Computational Logic Using Toehold Switches

Seungdo Choi, Geonhu Lee and Jongmin Kim *

Department of Life Sciences, Pohang University of Science and Technology, 77 Cheongam-ro, Pohang 37673, Gyeongbuk, Korea; choisd@postech.ac.kr (S.C.); kunhu0213@postech.ac.kr (G.L.)
* Correspondence: jongmin.kim@postech.ac.kr; Tel.: +82-54-279-2322

Citation: Choi, S.; Lee, G.; Kim, J. Cellular computational logic using toehold switches. *Int. J. Mol. Sci.* **2022**, *12*, 4265. https://doi.org/10.3390/ijms23084265

Academic Editors: Vaclav Brazda and Richard Bowater

Received: 8 March 2022
Accepted: 10 April 2022
Published: 12 April 2022

Publisher's Note: MDPI stays neutral with regard to jurisdictional claims in published maps and institutional affiliations.

Copyright: © 2022 by the authors. Submitted for possible open access publication under the terms and conditions of the Creative Commons Attribution (CC BY) license (https://creativecommons.org/licenses/by/4.0/).

Supplementary Tables

Supplementary Table S1. NUPACK scripts used in design steps. NUPACK version 4.0.0.27 was used to perform the incorporation of NIMPLY gate switches and the design of XOR gate with antisense RNAs. To note, NUPACK software was operated on Jupyter lab 3.3.2 in a local environment. The users are encouraged to explore other options for their designs. For example, the 5′ overhang of the trigger RNA and the 3′ overhang of the antisense RNA were disposable for gate function and the removal of bulge could be favorable to produce improved function.

Source Code
**Design_2OR_v4.0.0.27**

```
# Import NUPACK Python module
from nupack import *

# Specify Domains
# Switch Domains
S1 = Domain('GGGATTGAATATGATAGAAGTTTAGTAGTAGACAATAGAACAGAGGAGATATTGATGAC-
TACTAAACTA',name='S1')
LK = Domain('N9',name='LK')
S2 = Domain('ACTGATTTGAATACACTGCTTCGTTCACGATTCAGAACAGAGGAGATGAATATGGAACGAA-
GCAGA',name='S2')
UniLK = Domain('AACCTGGCGGCAGCGCAAAAG',name='UniLK')

# Trigger Domains
T1    = Domain('ACTACTAAACTTCTATCATATTCAAT',name='T1')
T2    = Domain('GAACGAAGCAGTGTATTCAAATCAGT',name='T2')

# Specify Strands
SW    = TargetStrand([S1,LK,S2,UniLK], name='SW')
TR1 = TargetStrand([T1], name='TR1')
TR2 = TargetStrand([T2], name='TR2')

# Specify Complexes
SWc       = TargetComplex([SW],
'.................((((((((((...(((((............)))))...))))))))))........................((((((((((...(((((............)))))...))))))))))......................',
name='SWc')
TR1c      = TargetComplex([TR1], '..........................', name='TR1c')
TR2c      = TargetComplex([TR2], '..........................', name='TR2c')
SW_TR1 = TargetComplex([SW,TR1],
'...((((((((((((((((((((((((...(((((............))))).......................................((((((((((...(((((............)))))...))))))))))......................+)))))))))))))
))))))))))))', name='SW_TR1')
SW_TR2 = TargetComplex([SW,TR2],
'.................((((((((((...(((((............)))))...))))))))))..........((((((((((((((((((((((((...(((((............)))))..................................+)))))))))))))
))))))))))))', name='SW_TR2')

# Specify Tubes
#Tube1= Switch-Trigger interaction
Tube1 = TargetTube(on_targets={SWc: 1e-8, TR1c: 1e-8, TR2c: 1e-8, SW_TR1: 1e-6, SW_TR2: 1e-6}, name='Tube1',
off_targets=SetSpec(max_size=3, include=[], exclude=[]))
# If the list of on/off target structures was needed, use print(Tube1.on_targets) or print(Tube1.off_targets)

# Run Test tube design
# Define Constraints
```

```
my_soft_constraints=[
    Pattern(['A4', 'C4', 'G4', 'U4', 'M6', 'K6', 'W6', 'S6', 'R6', 'Y6'])
]

# Run Test tube design
my_model = Model(material='rna06', ensemble='stacking', celsius=37, sodium=1.0, magnesium=0.0)
my_tubes = [Tube1]
my_design = tube_design(tubes=my_tubes,
    hard_constraints=[], soft_constraints=my_soft_constraints,
    defect_weights=None, options=None, model=my_model)

# Design order
trial=3 # number of Design order

my_jobs = my_design.launch(trials=trial, checkpoint='checkpoints', interval=600)
my_jobs.wait()
my_final_results = my_jobs.final_results()

print(my_final_results)
print(my_final_results[0])

file_name = 'Design_Result_ORgate_v4.txt'

data = open(file_name, 'w')
for i in range(trial):
    data.write("\n#############number %d#############\n" % (i+1))
    data.write(str(my_final_results[i]))
    data.write('\n')
```

Design_XOR_v4.0.0.27

```
# Import NUPACK Python module
from nupack import *

# Specify Domains
# Switch Domains
Switch = Domain('GGGATTGAATATGATAGAAGTTTAGTAGTACACAATAGAACAGAGGAGATATTGATGAC-
TACTAAACTAAAACACGCGACTGATTTGAATACACTGCTTCGTTCACGATTCAGAACAGAGGAGATGAATAT
GGAACGAAGCAGAAACCTGGCGGCAGCGCAAAAG',name='Switch')

# Trigger Domains - (Antisense Domains would be automatically defined with complementary funnction)
T1  = Domain('ACTACTAAACTTCTATCATATTCAAT',name='T1')
T15 = Domain('N16',name='T15')
T13 = Domain('N16',name='T13')
T2  = Domain('GAACGAAGCAGTGTATTCAAATCAGT',name='T2')
T25 = Domain('N16',name='T25')
T23 = Domain('N16',name='T23')

# Specify Strands
SW  = TargetStrand([Switch], name='SW')
TR1 = TargetStrand([T15,T1,T13], name='TR1')
```

```
TR2 = TargetStrand([T25,T2,T23], name='TR2')
AR1 = TargetStrand([~T13,~T1,~T15], name='AR1')
AR2 = TargetStrand([~T23,~T2,~T25], name='AR2')

# Specify Complexes
SWc      = TargetComplex([SW],
'.................((((((((((...(((((...........)))))...))))))))))........................((((((((((...(((((...........)))))...))))))))))......................',
name='SWc')
TR1c    = TargetComplex([TR1], '.........................................................', name='TR1c')
TR2c    = TargetComplex([TR2], '.........................................................', name='TR2c')
AR1c    = TargetComplex([AR1], '.........................................................', name='AR1c')
AR2c    = TargetComplex([AR2], '.........................................................', name='AR2c')
SW_TR1 = TargetComplex([SW,TR1],
'...((((((((((((((((((((((((((...(((((...........)))))..................................((((((((((...(((((...........)))))...))))))))))......................+................))
))))))))))))))))))))))))................', name='SW_TR1')
SW_TR2 = TargetComplex([SW,TR2],
'.................((((((((((.(((((((...........))))).))))))))))))..........((((((((((((((((((((((((((...(((((...........)))))...................................+................)
)))))))))))))))))))))))))................', name='SW_TR2')
TR1_AR1= TargetComplex([TR1,AR1],
'((((((((((((((.((((((((((((((((((((((((((.((((((((((((((+)))))))))))))).)))))))))))))))))))))))))).))))))))))))))', name='TR1_AR1')
TR2_AR2= TargetComplex([TR2,AR2],
'((((((((((((((.((((((((((((((((((((((((((.((((((((((((((+)))))))))))))).)))))))))))))))))))))))))).))))))))))))))', name='TR2_AR2')

# Specify Tubes
#Tube1= Switch-Trigger interaction
Tube1 = TargetTube(on_targets={SWc: 1e-8, TR1c: 1e-8, TR2c: 1e-8, SW_TR1: 1e-6, SW_TR2: 1e-6}, name='Tube1',
off_targets=SetSpec(max_size=2, include=[], exclude=[]))
#Tube2= Switch-Trigger-Antisense interaction
Tube2 = TargetTube(on_targets={SWc: 1e-8, TR1c: 1e-8, TR2c: 1e-8, AR1c: 1e-8, AR2c: 1e-8, TR1_AR1: 1e-6, TR2_AR2:
1e-6}, name='Tube2', off_targets=SetSpec(max_size=2, include=[], exclude=[SW_TR1,SW_TR2]))
# If the list of on/off target structures was needed, use print(Tube1.on_targets) or print(Tube1.off_targets)

# Run Test tube design
# Define Constraints
my_soft_constraints=[
    Pattern(['A4', 'C4', 'G4', 'U4', 'M6', 'K6', 'W6', 'S6', 'R6', 'Y6'])
]

# Run Test tube design
my_model = Model(material='rna06', ensemble='stacking', celsius=37, sodium=1.0, magnesium=0.0)
my_tubes = [Tube1,Tube2]
my_design = tube_design(tubes=my_tubes,
    hard_constraints=[], soft_constraints=my_soft_constraints,
    defect_weights=None, options=None, model=my_model)

# Design order
trial=100 # number of Design order

my_jobs = my_design.launch(trials=trial, checkpoint='checkpoints', interval=600)
my_jobs.wait()
my_final_results = my_jobs.final_results()
```

```
print(my_final_results)
print(my_final_results[0])

file_name = 'Design_Result_XORgate_v4.txt'

data = open(file_name, 'w')
for i in range(trial):
    data.write("\n#############number %d#############\n" % (i+1))
    data.write(str(my_final_results[i]))
    data.write('\n')
```

Supplementary Table S2. Free energy of MFE structure between On-/Off-target strands. Free energy of MFE structure was calculated with NUPACK 4.0.0.27. To note, parameter of rna06 was selected for calculation algorithm.

Name		Classification	ΔG of MFE Structure
Trigger 1	Antisense 1	On-target	-75.53 kcal/mol
Trigger 1	Antisense 2	Off-target	-8.76 kcal/mol
Trigger 2	Antisense 1	Off-target	-11.77 kcal/mol
Trigger 2	Antisense 2	On-target	-104.28 kcal/mol
Trigger 1	Trigger 2	Off-target	-14.79 kcal/mol
Antisense 1	Antisense 2	Off-target	-14.05 kcal/mol
AND trigger 1	AND trigger 2	On-target	-61.61 kcal/mol
AND trigger 1	Trigger 1	Off-target	-29.10 kcal/mol
AND trigger 1	Trigger 2	Off-target	-20.93 kcal/mol
AND trigger 1	Antisense 1	Off-target	-17.94 kcal/mol
AND trigger 1	Antisense 2	Off-target	-32.48 kcal/mol
AND trigger 2	Trigger 1	Off-target	-20.34 kcal/mol
AND trigger 2	Trigger 2	Off-target	-26.80 kcal/mol
AND trigger 2	Antisense 1	Off-target	-24.53 kcal/mol
AND trigger 2	Antisense 2	Off-target	-18.71 kcal/mol
NIMPLY trigger	NIMPLY antisense	On-target	-118.45 kcal/mol
NIMPLY trigger	Trigger 1	Off-target	-25.98 kcal/mol
NIMPLY trigger	Trigger 2	Off-target	-35.65 kcal/mol
NIMPLY trigger	Antisense 1	Off-target	-35.20 kcal/mol
NIMPLY trigger	Antisense 2	Off-target	-31.72 kcal/mol
NIMPLY antisense	Trigger 1	Off-target	-36.35 kcal/mol
NIMPLY antisense	Trigger 2	Off-target	-34.52 kcal/mol
NIMPLY antisense	Antisense 1	Off-target	-34.96 kcal/mol
NIMPLY antisense	Antisense 2	Off-target	-36.23 kcal/mol

Supplementary Table S3. Plasmids used in this study. Abbreviations are as follows: pT7 = T7 promoter, T7term = T7 terminator, CmR = chloramphenicol resistance gene, AmpR = ampicillin resistance gene, SpecR = spectinomycin resistance gene, KanR = kanamycin resistance gene. XOR switch and AND/NIMPLY switch were cloned into pACYCDUET and pCOLADUET plasmid, respectively. All the trigger and antisense RNAs were cloned into pCDFDUET, pET15b plasmid, respectively.

Name	Sequence
**NIMPLY complex**	
XOR switch	pT7–XOR switch–Linker-GFPmut3b_ASV-T7term-CmR-p15A origin-TetR-LacI
Trigger RNA	pT7-Trigger RNA-T7term-SpecR-CloDF origin-LacI
Antisense RNA	pT7-Antisense RNA-T7term-AmpR-pBR322 origin-LacI
**XOR gate**	
Trigger cassette	pT7-tetO-Trigger2-T7term-pT7-lacO-Trigger1-T7term-SpecR-CloDF origin-LacI
Antisense cassette	pT7-lacO-Antisense2-T7term-pT7-tetO-Antisense1-T7term-AmpR-pBR322 origin-LacI
**Half adder, half subtractor, and Feynman gate**	
AND/NIMPLY switch	pT7–AND/NIMPLY switch–Linker-mCherry-T7term-KanR-ColA origin-LacI

Supplementary Table S4. Examples of DNA plasmid sequences. Replication origin, *tetR* and *lacI* are reversely oriented to other elements.

Name (Architecture)	Sequence
XOR switch (pT7–XOR switch(RBS)–Linker-GFPmut3b_ASV-T7term– CmR–(cat Promoter)-p15A origin-back-bone-TetR-(J23106 promoter)-LacI-(Lac promoter))	TAATACGACTCACTATAGGGATTGAATATGATAGAAGTTTAGTAGTAGA-<br>CAATAGAACAGAG-<br>GAGATATTGATGACTACTAAACTAAAACACGCGACTGATTTGAATACACTG<br>CTTCGTTCACGATTCAAGAAAAGAACAGAGGAGATGAATATGGAACGAA-<br>GCAGAAAC-<br>CTGGCGGCAGCGCAAAAGATGCGTAAAGGAGAAGAACTTTTCACTGGAGT<br>TGTCCCAATTCTTGTTGAATTAGATGGTGATGTTAATGGG-<br>CACAAATTTTCTGTCAGTGGA-<br>GAGGGTGAAGGTGATGCAACATACGGAAAACTTACCCTTAAATTTATTTGC<br>ACTACTGGAAAACTACCTGTTCCGTGGCCAACACTTGTCAC-<br>TACTTTCGGTTATGGTGTTCAATGCTTTGCGAGATACCCAGATCACATGAAA<br>CAGCATGACTTTTTCAAGAGTGCCATGCCCGAAGGTTACGTACAGGAAA-<br>GAACTA-<br>TATTTTTCAAAGATGACGGGAACTACAAGACACGTGCTGAAGTCAAGTTTG<br>AAGGTGATACCCTTGTTAATAGAATCGAGTTAAAAGGTATTGATTTTAAA-<br>GAA-<br>GATGGAAACATTCTTGGACACAAATTGGAATACAACTATAACTCACACAA<br>TGTATACATCATGGCAGACAAACAAAA-<br>GAATGGAATCAAAGTTAACTTCAAAATTAGA-<br>CACAACATTGAAGATGGAAGCGTTCAACTAGCAGACCATTATCAACAAAA<br>TACTCCGATTGGCGATGGCCCTGTCCTTTTACCAGACAACCATTAC-<br>CTGTCCACACAATCTGCCCTTTCGAAAGATCCCAACGAAAAGAGAGACCA<br>CATGGTCCTTCTTGAGTTTGTAACCGCTGCTGGGATTACACATGG-<br>CATGGATGAACTATA-<br>CAAAAGGCCTGCAGCAAACGACGAAAACTACGCTGCATCAGTTTAATAAG<br>ATAAACCAGAGCGGCACGGCAAGCAGAGTATACGAGATTCGGTAGCCAC-<br>CGCTGAGCAA-<br>TAACTAGCATAACCCCTTGGGGCCTCTAAACGGGTCTTGAGGGGTTTTTTGG<br>GCGAGCTACTCGTTTGCGTAGAAGTAGTTGGCATTTGAGAAGCACAC-<br>GGTCACAC-<br>TGCTTCCGGTAGTCAATAAACCGGTAAACCAGCAATAGACATAAGCGGCT

	ATTTAACGACCCTGCCCTGAACCGACGACCGGGTCGAATTT-<br>GCTTTCGAATTTCTGCCATTCATCCGCTTATTATCACTTATTCAGGCGTAGC<br>AACCAGGCGTTTAAGGGCACCAATAACTGCCTTAAAAAAATTAC-<br>GCCCCGCCCTGCCAC-<br>TCATCGCAGTACTGTTGTAATTCATTAAGCATTCTGCCGACATGGAAGCCA<br>TCACAGACGGCATGATGAACCTGAATCGCCAGCGGCATCAGCACCTT-<br>GTCGCCTTGCG-<br>TATAATATTTGCCCATAGTGAAAACGGGGGCGAAGAAGTTGTCCATATTGG<br>CCACGTTTAAATCAAAACTGGTGAAACTCACCCAGGGATTGGCTGAGAC-<br>GAAAAACATATTCTCAATAAACCCTTTAGGGAAATAGGCCAGGTTTTCACC<br>GTAACACGCCACATCTTGCGAATATATGTGTAGAAACTGCCG-<br>GAAATCGTCGTGGTATTCAC-<br>TCCAGAGCGATGAAAACGTTTCAGTTTGCTCATGGAAAACGGTGTAACAA<br>GGGTGAACACTATCCCATATCACCAGCTCACCGTCTTTCATT-<br>GCCATACGGAACTCCG-<br>GATGAGCATTCATCAGGCGGGCAAGAATGTGAATAAAGGCCGGATAAAAC<br>TTGTGCTTATTTTTCTTTACGGTCTTTAAAAAGGCCGTAA-<br>TATCCAGCTGAAC-<br>GGTCTGGTTATAGGTACATTGAGCAACTGACTGAAATGCCTCAAAATGTTC<br>TTTACGATGCCATTGGGATATATCAACGGTGGTATATCCAGTGAT-<br>TTTTTTCTCCATTTTAGCTTCCTTAGCTCCTGAAAATCTCGATAACTCAAAAA<br>ATACGCCCGGTAGTGATCTTATTTCATTATGGTGAAAGTTGGAAC-<br>CTCTTAC-<br>GTGCCGATCAACGTCTCATTTTCGCCAAAAGTTGGCCCAGGGCTTCCCGGT<br>ATCAACAGGGACACCAGGAT-<br>TTATTTATTCTGCGAAGTGATCTTCCGTCACAGGTATTTATTCGGCGCAAAG<br>TGCGTCGGGTGATGCTGCCAACTTACTGATTTAGTGTATGATGGTGTTTTT-<br>GAGGTGCTCCAGTGGCTTCTGTTTCTATCAGCTGTCCCTCCTGTTCAGCTACT<br>GACGGGGTGGTGCGTAACGGCAAAAGCACCGCCG-<br>GACATCAGCGCTAGCGGAGTG-<br>TATACTGGCTTACTATGTTGGCACTGATGAGGGTGTCAGTGAAGTGCTTCAT<br>GTGGCAGGAGAAAAAAGGCTGCACCGGTGCGTCAGCAGAATATGTGATA-<br>CAGGATA-<br>TATTCCGCTTCCTCGCTCACTGACTCGCTACGCTCGGTCGTTCGACTGCGGC<br>GAGCGGAAATGGCTTACGAACGGGGCGGAGATTTCCTGGAAGATGCCAG-<br>GAAGA-<br>TACTTAACAGGGAAGTGAGAGGGCCGCGGCAAAGCCGTTTTTCCATAGGC<br>TCCGCCCCCCTGACAAGCATCACGAAATCTGACGCTCAAATCAG-<br>TGGTGGCGAAACCCGACAGGACTATAAAGATACCAGGCGTTTCCCCTGGC<br>GGCTCCCTCGTGCGCTCTCCTGTTCCTGCCTTTCGGTTTAC-<br>CGGTGTCATTCCGCTGTTATGGCCGCGTTTGTCTCATTCCACGCCTGACACT<br>CAGTTCCGGGTAGGCAGTTCGCTCCAAGCTGGACTGTATGCAC-<br>GAACCCCCCGTTCAG-<br>TCCGACCGCTGCGCCTTATCCGGTAACTATCGTCTTGAGTCCAACCCGGAA<br>AGACATGCAAAAGCACCACTGGCAGCAGCCACTGGTAATTGATTTAGAG-<br>GAGTTAGTCTT-<br>GAAGTCATGCGCCGGTTAAGGCTAAACTGAAAGGACAAGTTTTGGTGACT<br>GCGCTCCTCCAAGCCAGTTACCTCGGTTCAAAGAGTTGGTAGCTCAGA-<br>GAAC-<br>CTTCGAAAAACCGCCCTGCAAGGCGGTTTTTTCGTTTTCAGAGCAAGAGAT

	TACGCGCAGACCAAAACGATCTCAAGAAGATCATCTTATTAATCAGA-<br>TAAAATATTTCTA-<br>GATTTCAGTGCAATTTATCTCTTCAAATGTAGCACCTGAAGTCAGCCCCATA<br>CGATATAAGTTGTGAAGATCATCTTATTAATCAGATAAAATATTTCTAGAT-<br>TTCAGTG-<br>CAATTTATCTCTTCAAATGTAGCACCTGAAGTCAGCCCCATACGATATAAG<br>TTGTAATTCTCATGTTAGTCTCAAGACCCACTTTCACATTTAAGTT-<br>GTTTTTCTAATCCG-<br>CAGATGATCAATTCAAGGCCGAATAAGAAGGCTGGCTCTGCACCTTGGTG<br>ATCAAATAATTCGATAGCTTGTCGTAATAATGGCGGCATACTATCAGTAG-<br>TAGGTGTTTCCCTTTCTTCTTTAGCGACTTGATGCTCTTGATCTTCCAATACG<br>CAACCTAAAGTAAAATGCCCCACAGCGCTGAGTGCATA-<br>TAATGCATTCTCTAGTGAAAAAC-<br>CTTGTTGGCATAAAAAGGCTAATTGATTTTCGAGAGTTTCATACTGTTTTTCT<br>GTAGGCCGTGTACCTAAATGTACTTTTGCTCCATCGCGATGACTTAGTAAA-<br>GCACATCTAAAACTTTTAGCGTTATTACGTAAAAAATCTTGCCAGCTTTCCC<br>CTTCTAAAGGGCAAAAGTGAGTATGGTGCC-<br>TATCTAACATCTCAATGGCTAAGGCGTCGAG-<br>CAAAGCCCGCTTATTTTTTACATGCCAATACAATGTAGGCTGCTCTACACCT<br>AGCTTCTGGGCGAGTTTACGGGTTGTTAAACCTTCGATTCCGAC-<br>CTCATTAA-<br>GCAGCTCTAATGCGCTGTTAATCACTTTACTTTTATCTAAACGAGACATATC<br>TAACCTCCTTACTTTCCGCTTTATGGTGCGTCGACGCTAGCACTATACCTAG-<br>GACTGAGCTAGCCGTAAACTCACAATTCCATGGAACGGGCGGCCGCTGAC<br>TGGGTTGAAGGCTCTCAAGGGCATCGGTCGAGATCCCGGTGCCTAATGAG-<br>TGAGCTAACTTACATTAATTGCGTTGCGCTCACTGCCCGCTTTCCAGTCGGG<br>AAACCTGTCGTGCCAGCTGCATTAATGAATCGGCCAACGCGCGGGGA-<br>GAGGCGGTTTGCG-<br>TATTGGGCGCCAGGGTGGTTTTTCTTTTCACCAGTGAGACGGGCAACAGCT<br>GATTGCCCTTCACCGCCTGGCCCTGAGAGAGTTGCAGCAAGCGGTCCAC-<br>GCTGGTTT-<br>GCCCCAGCAGGCGAAAATCCTGTTTGATGGTGGTTAACGGCGGGATATAA<br>CATGAGCTGTCTTCGGTATCGTCGTATCCCACTACCGAGATGTCCGCAC-<br>CAACGCG-<br>CAGCCCGGACTCGGTAATGGCGCGCATTGCGCCCAGCGCCATCTGATCGTT<br>GGCAACCAGCATCGCAGTGGGAACGATGCCCTCATTCAGCATTT-<br>GCATGGTTTGTTGAAAAC-<br>CGGACATGGCACTCCAGTCGCCTTCCCGTTCCGCTATCGGCTGAATTTGATT<br>GCGAGTGAGATATTTATGCCAGCCAGCCAGACGCAGACGCGCCGAGA-<br>CAGAACTTAATGGGCCCGCTAACAGCGCGATTTGCTGGTGACCCAATGCG<br>ACCAGATGCTCCACGCCCAGTCGCGTACCGTCTTCATGGGAGAAAATAA-<br>TACTGTT-<br>GATGGGTGTCTGGTCAGAGACATCAAGAAATAACGCCGGAACATTAGTGC<br>AGGCAGCTTCCACAGCAATGGCATCCTGGTCATCCAGCGGA-<br>TAGTTAATGATCAGCCCAC-<br>TGACGCGTTGCGCGAGAAGATTGTGCACCGCCGCTTTACAGGCTTCGACGC<br>CGCTTCGTTCTACCATCGACACCACCACGCTGGCACCCAGTT-<br>GATCGGCGCGAGAT-<br>TTAATCGCCGCGACAATTTGCGACGGCGCGTGCAGGGCCAGACTGGAGGT<br>GGCAACGCCAATCAGCAACGACTGTTTGCCCGCCAGTTGTTGTGCCAC-

	GCGGTTGG-<br>GAATGTAATTCAGCTCCGCCATCGCCGCTTCCACTTTTTCCCGCGTTTTCGC<br>AGAAACGTGGCTGGCCTGGTTCACCACGCGGGAAACGGTCTGATAA-<br>GAGACACCGGCAT-<br>ACTCTGCGACATCGTATAACGTTACTGGTTTCACATTCACCACCCTGAATTG<br>ACTCTCTTCCGGGCGCTATCATGCCATACCGCGAAAGGTTTT-<br>GCGCCATTCGATGGTGTCCGGGATCTCGACGCTCTCCCTTATGCTAGTAAAT<br>TCGCGTTTCTACGGTAGCCGGGCGC
Trigger cassette (pT7–Trigger2–T7term–SpecR–(Bla Promoter)–CloDF13 origin–backbone-LacI-(Lac promoter))	TAATACGACTCACTATAGGGAACGAAGCAGTGTATTCAAATCAGTAAAA-<br>GAAAAGAGTTGG-<br>TAGCATAACCCCTTGGGGCCTCTAAACGGGTCTTGAGGGGTTTTTTGCACTT<br>TCGCAAAACTGAGAGCTCACGTGTAGGCATTTGAGAAGCACAC-<br>GGTCACACTGCTTCCGG-<br>TAGTCAATAAACGCTTCTCAAATGCCTGAGGTTTCAGGCGCGGCGTAGACA<br>CCAGACAAACGGCTATCACGGTAAACCAGCAATAGACATAAGCGGC-<br>TATTTAAC-<br>GACCCTGCCCTGAACCGACGACCGGGTCATCGTGGCCGGATCTTGCGGCCC<br>CTCGGCTTGAACGAATTGTTAGACATTATTTGCCGACTACCTT-<br>GGTGATCTCGCCTTTCAC-<br>GTAGTGGACAAATTCTTCCAACTGATCTGCGCGCGAGGCCAAGCGATCTTC<br>TTCTTGTCCAAGATAAGCCTGTCTAGCTTCAAGTATGACGGGCTGA-<br>TACTGGGCCGG-<br>CAGGCGCTCCATTGCCCAGTCGGCAGCGACATCCTTCGGCGCGATTTTGCC<br>GGTTACTGCGCTGTACCAAATGCGGGACAACGTAAGCAC-<br>TACATTTCGCTCATCGCCAGCCCAGTCGGGCGGCGAGTTCCATAGCGTTAA<br>GGTTTCATTTAGCGCCTCAAATAGATCCTGTTCAGGAACCGGATCAAA-<br>GAG-<br>TTCCTCCGCCGCTGGACCTACCAAGGCAACGCTATGTTCTCTTGCTTTTGTC<br>AGCAAGATAGCCAGATCAATGTCGATCGTGGCTGGCTCGAAGATACCTG-<br>CAAGAATGTCATT-<br>GCGCTGCCATTCTCCAAATTGCAGTTCGCGCTTAGCTGGATAACGCCACGG<br>AATGATGTCGTCGTGCACAACAATGGTGACTTCTACAGCGCGGA-<br>GAATCTCGCTCTCTCCAGGGGAAGCCGAAGTTTCCAAAAGGTCGTTGATCA<br>AAGCTCGCCGCGTTGTTTCATCAAGCCTTACGGTCACCGTAACCAG-<br>CAAATCAATATCAC-<br>TGTGTGGCTTCAGGCCGCCATCCACTGCGGAGCCGTACAAATGTACGGCCA<br>GCAACGTCGGTTCGAGATGGCGCTCGATGACGCCAACTACCTCTGA-<br>TAGTTGAGTCGA-<br>TACTTCGGCGATCACCGCTTCCCTCATACTCTTCCTTTTTCAATATTATTGAA<br>GCATTTATCAGGGTTATTGTCTCATGAGCGGATACATATTTGAATGTATTTA-<br>GAAAAA-<br>TAAACAAATAGCTAGCTCACTCGGTCGCTACGCTCCGGGCGTGAGACTGCG<br>GCGGGCGCTGCGGACACATACAAAGTTACCCACAGATTCCGTGGATAA-<br>GCAGGG-<br>GACTAACATGTGAGGCAAAACAGCAGGGCCGCGCCGGTGGCGTTTTTCCA<br>TAGGCTCCGCCCTCCTGCCAGAGTTCACATAAACAGACGCTTTTCCGGTG-<br>CATCTGTGG-<br>GAGCCGTGAGGCTCAACCATGAATCTGACAGTACGGGCGAAACCCGACAG<br>GACTTAAAGATCCCCACCGTTTCCGGCGGGTCGCTCCCTCTT-<br>GCGCTCTCCTGTTCCGACCCTGCCGTTTACCGGATACCTGTTCCGCCTTTCTC

	CCTTACGGGAAGTGTGGCGCTTTCTCATAGCTCACACACTGG-<br>TATCTCGGCTCGGTG-<br>TAGGTCGTTCGCTCCAAGCTGGGCTGTAAGCAAGAACTCCCCGTTCAGCCC<br>GACTGCTGCGCCTTATCCGGTAACTGTTCACTTGAGTCCAACCCGGAAAA-<br>GCACGGTAAAAC-<br>GCCACTGGCAGCAGCCATTGGTAACTGGGAGTTCGCAGAGGATTTGTTTAG<br>CTAAACACGCGGTTGCTCTTGAAGTGTGCGCCAAAGTCCGGCTACACTG-<br>GAAGGACAGATTT-<br>GGTTGCTGTGCTCTGCGAAAGCCAGTTACCACGGTTAAGCAGTTCCCCAAC<br>TGACTTAACCTTCGATCAAACCAC-<br>CTCCCCAGGTGGTTTTTTCGTTTACAGGGCAAAAGAT-<br>TACGCGCAGAAAAAAAGGATCTCAAGAAGATCCTTTGATCTTTTCTACTGA<br>ACCGCTCTAGATTTCAGTGCAATTTATCTCTTCAAATGTAGCAC-<br>CTGAAGTCAGCCCCATACGATATAAGTTGTAATTCTCATGTTAGTCATGCCC<br>CGCGCCCACCGGAAGGAGCTGACTGGGTTGAAGGCTCTCAAGGG-<br>CATCGGTCGA-<br>GATCCCGGTGCCTAATGAGTGAGCTAACTTACATTAATTGCGTTGCGCTCA<br>CTGCCCGCTTTCCAGTCGGGAAACCTGTCGTGCCAGCTG-<br>CATTAATGAATCGGCCAAC-<br>GCGCGGGGAGAGGCGGTTTGCGTATTGGGCGCCAGGGTGGTTTTTCTTTTC<br>ACCAGTGAGACGGGCAACAGCTGATTGCCCTTCACCGCCTGGCCCTGAGA-<br>GAGTTGCAGCAA-<br>GCGGTCCACGCTGGTTTGCCCCAGCAGGCGAAAATCCTGTTTGATGGTGGT<br>TAACGGCGGGATATAACATGAGCTGTCTTCGGTATCGTCGTATCCCACTAC-<br>CGAGATGTCCG-<br>CACCAACGCGCAGCCCGGACTCGGTAATGGCGCGCATTGCGCCCAGCGCC<br>ATCTGATCGTTGGCAACCAGCATCGCAGTGGGAACGATGCCCTCATTCAG-<br>CATTT-<br>GCATGGTTTGTTGAAAACCGGACATGGCACTCCAGTCGCCTTCCCGTTCCG<br>CTATCGGCTGAATTTGATTGCGAGTGAGATATTTATGCCAGCCAGCCAGAC-<br>GCAGAC-<br>GCGCCGAGACAGAACTTAATGGGCCCGCTAACAGCGCGATTTGCTGGTGA<br>CCCAATGCGACCAGATGCTCCACGCCCAGTCGCGTACCGTCTTCATGGGA-<br>GAAAATAA-<br>TACTGTTGATGGGTGTCTGGTCAGAGACATCAAGAAATAACGCCGGAACA<br>TTAGTGCAGGCAGCTTCCACAGCAATGGCATCCTGGTCATCCAGCGGA-<br>TAGTTAATGATCAGCCCACTGACGCGTTGCGCGAGAAGATTGTGCACCGCC<br>GCTTTACAGGCTTCGACGCCGCTTCGTTCTACCATCGACACCACCAC-<br>GCTGGCACCCAGTT-<br>GATCGGCGCGAGATTTAATCGCCGCGACAATTTGCGACGGCGCGTGCAGG<br>GCCAGACTGGAGGTGGCAACGCCAATCAGCAACGACTGTTTGCCCGCCAG-<br>TTGTTGTGCCAC-<br>GCGGTTGGGAATGTAATTCAGCTCCGCCATCGCCGCTTCCACTTTTTCCCGC<br>GTTTTCGCAGAAACGTGGCTGGCCTGGTTCACCACGCGGGAAAC-<br>GGTCTGATAAGAGACAC-<br>CGGCATACTCTGCGACATCGTATAACGTTACTGGTTTCACATTCACCACCCT<br>GAATTGACTCTCTTCCGGGCGCTATCATGCCATACCGCGAAAGGTTTT-<br>GCGCCATTCGATGGTGTCCGGGATCTCGACGCTCTCCCTTATGAGTGATAG<br>CCGTTTGTCTGGTGTCTACGCCGCGCGGGCTAACTGTC
Antisense cassette (pT7–Anti-<br>sense2–T7term-(Bla Promoter)–	TAATACGACTCACTATAGGGCTAGCTCTTTTCTTTTACTGATTTGAATACAC-<br>TGCTTCGTTCTCTACCCTTTCCCTTCTAGCATAACCCCTTGGGGCCTCTAAAC

AmpR-PBR322 origin–backbone-LacI-(Lac promoter))	GGGTCTTGAGGGGTTTTTTGCTGAAAGGAGGAACTATATCCGGATATCCCG-<br>CAA-<br>GAGGCCCGGCAGTACCGGCATAACCAAGCCTATGCCTACAGCATCCAGGG<br>TGACGGTGCCGAGGATGACGATGAGCGCATTGTTAGATCCGGATA-<br>TAGTTCCTCCTTTCAGGCGCAACGAAAGCCAGATTTTCATACACGGTGCCT<br>GACTGCGTTAGCAATTTAACTGTGATAAACTACCGCATTAAA-<br>GCTTATCGATGATAA-<br>GCTGTCAAACATGAGAATTCTTGAAGACGAAAGGGCCTCGTGATACGCCT<br>ATTTTTATAGGTTAATGTCATGATAATAATGGTTTCTTAGACGTCAGGTGG-<br>CACTTTTCGGG-<br>GAAATGTGCGCGGAACCCCTATTTGTTTATTTTTCTAAATACATTCAAATAT<br>GTATCCGCTCATGAGACAATAACCCTGATAAATGCTTCAATAA-<br>TATTGAAAAAGGAAGAG-<br>TATGAGTATTCAACATTTCCGTGTCGCCCTTATTCCCTTTTTTGCGGCATTTT<br>GCCTTCCTGTTTTTGCTCACCCAGAAACGCTGGTGAAAGTAAAA-<br>GATGCTGAAGATCAGTT-<br>GGGTGCACGAGTGGGTTACATCGAACTGGATCTCAACAGCGGTAAGATCC<br>TTGAGAGTTTTCGCCCCGAAGAACGTTTTCCAATGATGAGCAC-<br>TTTTAAAGTTCTGC-<br>TATGTGGCGCGGTATTATCCCGTGTTGACGCCGGGCAAGAGCAACTCGGTC<br>GCCGCATACACTATTCTCAGAATGACTTGGTTGAGTACTCACCAG-<br>TCACAGAAAA-<br>GCATCTTACGGATGGCATGACAGTAAGAGAATTATGCAGTGCTGCCATAAC<br>CATGAGTGATAACACTGCGGCCAACTTACTTCTGACAACGATCGGAGGAC-<br>CGAAGGAGCTAACCGCTTTTTTGCACAACATGGGGGATCATGTAACTCGCC<br>TTGATCGTTGGGAACCGGAGCTGAATGAAGCCATACCAAACGAC-<br>GAGCGTGACACCAC-<br>GATGCCTGCAGCAATGGCAACAACGTTGCGCAAACTATTAACTGGCGAAC<br>TACTTACTCTAGCTTCCCGGCAACAATTAATAGACTGGATGGAGGCGGA-<br>TAAAGTTGCAG-<br>GACCACTTCTGCGCTCGGCCCTTCCGGCTGGCTGGTTTATTGCTGATAAATC<br>TGGAGCCGGTGAGCGTGGGTCTCGCGGTATCATTGCAGCAC-<br>TGGGGCCAGATGGTAA-<br>GCCCTCCCGTATCGTAGTTATCTACACGACGGGGAGTCAGGCAACTATGGA<br>TGAACGAAATAGACAGATCGCTGAGATAGGTGCCTCACTGATTAAGCATT-<br>GG-<br>TAACTGTCAGACCAAGTTTACTCATATATACTTTAGATTGATTTAAAACTTC<br>ATTTTTAATTTAAAAGGATCTAGGTGAAGATCCTTTTTGATAATCTCATGAC-<br>CAAAATCCCTTAACGTGAGTTTTCGTTCCACTGAGCGTCAGACCCCGTAGA<br>AAAGATCAAAGGATCTTCTTGAGATCCTTTTTTTCTGCGCG-<br>TAATCTGCTGCTT-<br>GCAAACAAAAAAACCACCGCTACCAGCGGTGGTTTGTTTGCCGGATCAAG<br>AGCTACCAACTCTTTTTCCGAAGGTAACTGGCTTCAGCAGAGCGCAGA-<br>TACCAAA-<br>TACTGTCCTTCTAGTGTAGCCGTAGTTAGGCCACCACTTCAAGAACTCTGTA<br>GCACCGCCTACATACCTCGCTCTGCTAATCCTGTTACCAG-<br>TGGCTGCTGCCAGTGGCGA-<br>TAAGTCGTGTCTTACCGGGTTGGACTCAAGACGATAGTTACCGGATAAGGC<br>GCAGCGGTCGGGCTGAACGGGGGGTTCGTGCACACAGCCCAGCTT-<br>GGAGCGAACGAC-<br>CTACACCGAACTGAGATACCTACAGCGTGAGCTATGAGAAAGCGCCACGC

	TTCCCGAAGGGAGAAAGGCGGACAGGTATCCGGTAAGCGGCAGGGTCG-<br>GAACAGGAGAGCG-<br>CACGAGGGAGCTTCCAGGGGGAAACGCCTGGTATCTTTATAGTCCTGTCGG<br>GTTTCGCCACCTCTGACTTGAGCGTCGATTTTT-<br>GTGATGCTCGTCAGGGGGGCGGAGCC-<br>TATGGAAAAACGCCAGCAACGCGGCCTTTTTACGGTTCCTGGCCTTTTGCTG<br>GCCTTTTGCTCACATGTTCTTTCCTGCGTTATCCCCTGATTCTGTGGATAAC-<br>CGTATTAC-<br>CGCCTTTGAGTGAGCTGATACCGCTCGCCGCAGCCGAACGACCGAGCGCA<br>GCGAGTCAGTGAGCGAGGAAGCGGAAGAGCGCCTGATGCGG-<br>TATTTTCTCCTTAC-<br>GCATCTGTGCGGTATTTCACACCGCATATATGGTGCACTCTCAGTACAATCT<br>GCTCTGATGCCGCATAGTTAAGCCAGTATACACTCCGCTATCGCTAC-<br>GTGACTGGGTCATGGCTGCGCCCCGACACCCGCCAACACCCGCTGACGCG<br>CCCTGACGGGCTTGTCTGCTCCCGGCATCCGCTTACAGACAAGCTGTGAC-<br>CGTCTCCGG-<br>GAGCTGCATGTGTCAGAGGTTTTCACCGTCATCACCGAAACGCGCGAGGC<br>AGCTGCGGTAAAGCTCATCAGCGTGGTCGTGAAGCGAT-<br>TCACAGATGTCTGCCTGTTCATCCGCGTCCAGCTCGTTGAGTTTCTCCAGAA<br>GCGTTAATGTCTGGCTTCTGATAAA-<br>GCGGGCCATGTTAAGGGCGGTTTTTTCCTGTTT-<br>GGTCACTGATGCCTCCGTGTAAGGGGGATTTCTGTTCATGGGGGTAATGAT<br>ACCGATGAAACGAGAGAGGATGCTCACGATAC-<br>GGGTTACTGATGATGAACATGCCCGGTTACTGGAACGTTGTGAGGGTAAAC<br>AACTGGCGGTATGGATGCGGCGGGACCAGAGAAAAATCAC-<br>TCAGGGTCAATGCCAGCGCTTCGTTAATACAGATGTAGGTGTTCCACAGGG<br>TAGCCAGCAGCATCCTGCGATGCAGATCCGGAACATAATGGTG-<br>CAGGGCGCTGACTTCCGCGTTTCCAGACTTTACGAAACACGGAAACCGAA<br>GACCATTCATGTTGTTGCTCAGGTCGCAGACGTTTTGCAGCAGCAG-<br>TCGCTTCAC-<br>GTTCGCTCGCGTATCGGTGATTCATTCTGCTAACCAGTAAGGCAACCCCGC<br>CAGCCTAGCCGGGTCCTCAACGACAGGAGCACGATCATGCG-<br>CACCCGTGGCCAGGACCCAAC-<br>GCTGCCCGAGATGCGCCGCGTGCGGCTGCTGGAGATGGCGGACGCGATGG<br>ATATGTTCTGCCAAGGGTTGGTTTGCGCATTCACAGTTCTCCGCAAGAATT-<br>GATTGGCTCCAATTCTTGGAGTGGTGAATCCGTTAGCGAGGTGCCGCCGGC<br>TTCCATTCAGGTCGAGGTGGCCCGGCTCCATGCACCGCGACGCAAC-<br>GCGGGGAGGCAGA-<br>CAAGGTATAGGGCGGCGCCTACAATCCATGCCAACCCGTTCCATGTGCTCG<br>CCGAGGCGGCATAAATCGCCGTGACGATCAGCGGTCCAG-<br>TGATCGAAGTTAGGCTGGTAA-<br>GAGCCGCGAGCGATCCTTGAAGCTGTCCCTGATGGTCGTCATCTACCTGCC<br>TGGACAGCATGGCCTGCAACGCGGGCATCCCGATGCCGCCGGAAGCGA-<br>GAA-<br>GAATCATAATGGGGAAGGCCATCCAGCCTCGCGTCGCGAACGCCAGCAAG<br>ACGTAGCCCAGCGCGTCGGCCGCCATGCCGGCGA-<br>TAATGGCCTGCTTCTCGCCGAAACGTTT-<br>GGTGGCGGGACCAGTGACGAAGGCTTGAGCGAGGGCGTGCAAGATTCCGA<br>ATACCGCAAGCGACAGGCCGATCATCGTCGCGCTCCAGCGAAA-<br>GCGGTCCTCGCCGAAAATGACCCAGAGCGCTGCCGGCACCTGTCCTACGA

	GTTGCATGATAAAGAAGACAGTCATAAGTGCGGCGACGA-<br>TAGTCATGCCCCGCGCCCACCG-<br>GAAGGAGCTGACTGGGTTGAAGGCTCTCAAGGGCATCGGTCGAGATCCCG<br>GTGCCTAATGAGTGAGCTAACTTACATTAATTGCGTTGCGCTCAC-<br>TGCCCGCTTTCCAG-<br>TCGGGAAACCTGTCGTGCCAGCTGCATTAATGAATCGGCCAACGCGCGGG<br>GAGAGGCGGTTTGCGTATTGGGCGCCAGGGTGGTTTTTCTTTTCACCAG-<br>TGAGACGGG-<br>CAACAGCTGATTGCCCTTCACCGCCTGGCCCTGAGAGAGTTGCAGCAAGCG<br>GTCCACGCTGGTTTGCCCCAGCAGGCGAAAATCCTGTTT-<br>GATGGTGGTTAACGGCGGGATA-<br>TAACATGAGCTGTCTTCGGTATCGTCGTATCCCACTACCGAGATATCCGCA<br>CCAACGCGCAGCCCGGACTCGGTAATGGCGCGCATT-<br>GCGCCCAGCGCCATCTGATCGTTGG-<br>CAACCAGCATCGCAGTGGGAACGATGCCCTCATTCAGCATTTGCATGGTTT<br>GTTGAAAACCGGACATGGCACTCCAGTCGCCTTCCCGTTCCGC-<br>TATCGGCTGAATTT-<br>GATTGCGAGTGAGATATTTATGCCAGCCAGCCAGACGCAGACGCGCCGAG<br>ACAGAACTTAATGGGCCCGCTAACAGCGCGATTT-<br>GCTGGTGACCCAATGCGAC-<br>CAGATGCTCCACGCCCAGTCGCGTACCGTCTTCATGGGAGAAAATAATACT<br>GTTGATGGGTGTCTGGTCAGAGACATCAAGAAATAACGCCGGAACATTAG-<br>TG-<br>CAGGCAGCTTCCACAGCAATGGCATCCTGGTCATCCAGCGGATAGTTAATG<br>ATCAGCCCACTGACGCGTTGCGCGAGAAGATTGTGCAC-<br>CGCCGCTTTACAGGCTTCGAC-<br>GCCGCTTCGTTCTACCATCGACACCACCACGCTGGCACCCAGTTGATCGGC<br>GCGAGATTTAATCGCCGCGACAATTTGCGACGGCGCGTG-<br>CAGGGCCAGACTGGAGGTGG-<br>CAACGCCAATCAGCAACGACTGTTTGCCCGCCAGTTGTTGTGCCACGCGGT<br>TGGGAATGTAATTCAGCTCCGCCATCGCCGCTTCCAC-<br>TTTTTCCCGCGTTTTCGCAGAAAC-<br>GTGGCTGGCCTGGTTCACCACGCGGGAAACGGTCTGATAAGAGACACCGG<br>CATACTCTGCGACATCGTATAACGTTACTGGTTTCACATTCAC-<br>CACCCTGAATT-<br>GACTCTCTTCCGGGCGCTATCATGCCATACCGCGAAAGGTTTTGCGCCATTC<br>GATGGTGTCCGGGATCTCGACGCTCTCCCTTATGCGACTCCTGCATTAG-<br>GAAGCAGCCCAG-<br>TAGTAGGTTGAGGCCGTTGAGCACCGCCGCCGCAAGGAATGGTGCATGCA<br>AGGAGATGGCGCCCAACAGTCCCCCGGCCACGGGGCCTGCCAC-<br>CATACCCACGCCGAAACAA-<br>GCGCTCATGAGCCCGAAGTGGCGAGCCCGATCTTCCCCATCGGTGATGTCC<br>GCGAAAT
AND switch (pT7–AND switch(RBS)–Linker–mCherry–T7term–KanR–(Bla Promoter)–ColA origin-backbone-LacI-(Lac promoter))	TAATACGACTCACTATAGGGACTACTATTGATTACAC-<br>GCTTTACTTCGAAATTCATAATGAACAGAGGAGATATGAAATGCGAAGTA<br>AAGCGAACCTGGCGGCAGCGCAAAAGATGCGTAAAGTGAG-<br>CAAGGGCGAAGAAGA-<br>TAACATGGCCATCATCAAGGAGTTCATGCGCTTCAAGGTTCACATGGAGGG<br>CTCCGTGAACGGCCACGAGTTCGA-<br>GATCGAGGGCGAGGGCGAGGGCCGCCCCTACGAGGG-<br>CACCCAGACCGCCAAGCTGAAGGTGACCAAGGGTGGCCCCCTGCCCTTCG

	CCTGGGACATCCTGTCCCCTCAGTTCATGTACGGCTCCAAGGCCTAC-<br>GTGAA-<br>GCACCCCGCCGACATCCCCGACTACTTGAAGCTGTCCTTCCCCGAGGGCTT<br>CAAGTGGGAGCGCGTGATGAACTTCGAGGACGGCGGCGTGGTGAC-<br>CGTGACCCAG-<br>GACTCCTCCCTGCAAGACGGCGAGTTCATCTACAAGGTGAAGCTGCGCGG<br>CACCAACTTCCCCTCCGACGGCCCCGTAATGCAGAAGAAGAC-<br>TATGGGCTGG-<br>GAGGCCTCCTCCGAGCGGATGTACCCCGAGGACGGCGCGCTGAAGGGCGA<br>GATCAAGCAGAGGCTGAAGCTGAAGGACGGCGGCCACTACGAC-<br>GCTGAGGTCAAGACCAC-<br>CTACAAGGCCAAGAAGCCCGTGCAACTGCCCGGCGCGTACAACGTCAACA<br>TCAAGTTGGACATCACCTCCCACAACGAGGACTACACCATCGTGGAACAG-<br>TACGAAC-<br>GCGCCGAGGGCCGCCACTCCACCGGCGGCATGGACGAGCTGTACAAGTAA<br>TAAAACAGTCGAGCCCAGCGTGGTTAAACACTCTAGCATAACCCCTT-<br>GGGGCCTCTAAAC-<br>GGGTCTTGAGGGGTTTTTTGCTGAAACCTCAGGCATTTGAGAAGCACACGG<br>TCACACTGCTTCCGGTAGTCAATAAACCGGTAAACCAGCAATAGACATAA-<br>GCGGC-<br>TATTTAACGACCCTGCCCTGAACCGACGACAAGCTGACGACCGGGTCTCCG<br>CAAGTGGCACTTTTCGGGGAAATGTGCGCGGAACCCCTATTT-<br>GTTTATTTTTCTAAATACATTCAAATATGTATCCGCTCATGAATTAATTCTTA<br>GAAAAACTCATCGAGCATCAAATGAAACTGCAATTTATTCATATCAGGAT-<br>TATCAATAC-<br>CATATTTTTGAAAAAGCCGTTTCTGTAATGAAGGAGAAAACTCACCGAGGC<br>AGTTCCATAGGATGGCAAGATCCTGGTATCGGTCTGCGAT-<br>TCCGACTCGTCCAACATCAATACAACCTATTAATTTCCCCTCGTCAAAAAT<br>AAGGTTATCAAGTGAGAAATCACCATGAGTGACGACTGAATCCGGTGA-<br>GAATGG-<br>CAAAAGTTTATGCATTTCTTTCCAGACTTGTTCAACAGGCCAGCCATTACGC<br>TCGTCATCAAAATCACTCGCATCAACCAAAC-<br>CGTTATTCATTCGTGATTGCGCCTGAGCGA-<br>GACGAAATACGCGGTCGCTGTTAAAAGGACAATTACAAACAGGAATCGAA<br>TGCAACCGGCGCAGGAACACTGCCAGCGCATCAACAATATTTTCAC-<br>CTGAATCAGGA-<br>TATTCTTCTAATACCTGGAATGCTGTTTTCCCGGGGATCGCAGTGGTGAGTA<br>ACCATGCATCATCAGGAGTACGGATAAAATGCTTGATGGTCGGAA-<br>GAGGCATAAATTCCGTCAGCCAGTTTAGTCTGACCATCTCATCTGTAACAT<br>CATTGGCAACGCTACCTTTGCCATGTTTCAGAAACAACTCTGGCG-<br>CATCGGGCTTCCCATACAATCGATAGATTGTCGCACCTGATTGCCCGACAT<br>TATCGCGAGCCCATTTATACCCATATAAATCAGCATCCATGTT-<br>GGAATTTAATCGCGGCCTA-<br>GAGCAAGACGTTTCCCGTTGAATATGGCTCATACTCTTCCTTTTTCAATATT<br>ATTGAAGCATTTATCAGGGTTATTGTCTCATGAGCGGATACATATTT-<br>GAATGTATTTA-<br>GAAAAATAAACAAATAGGCATGCTAGCGCAGAAACGTCCTAGAAGATGC<br>CAGGAGGATACTTAGCAGAGAGACAATAAGGCCGGAGCGAA-<br>GCCGTTTTTCCA-<br>TAGGCTCCGCCCCCCTGACGAACATCACGAAATCTGACGCTCAAATCAGTG

	GTGGCGAAACCCGACAGGACTATAAAGATAC-<br>CAGGCGTTTCCCCCTGATGGCTCCCTCTT-<br>GCGCTCTCCTGTTCCCGTCCTGCGGCGTCCGTGTTGTGGTGGAGGCTTTACC<br>CAAATCACCACGTCCCGTTCCGTGTAGACAGTTCGCTCCAA-<br>GCTGGGCTGTGTGCAA-<br>GAACCCCCCGTTCAGCCCGACTGCTGCGCCTTATCCGGTAACTATCATCTTG<br>AGTCCAACCCGGAAAGACACGACAAAACGCCACTGGCAGCAGCCATTGG-<br>TAACTGAGAATT-<br>AGTGGATTTAGATATCGAGAGTCTTGAAGTGGTGGCCTAACAGAGGCTACA<br>CTGAAAGGACAGTATTTGGTATCTGCGCTCCACTAAAGCCAGTTAC-<br>CAGGTTAAGCAG-<br>TTCCCCAACTGACTTAACCTTCGATCAAACCGCCTCCCCAGGCGGTTTTTTC<br>GTTTACAGAGCAGGAGATTACGACGATCGTAAAAGGATCTCAAGAA-<br>GATCCTTTACGGAT-<br>TCCCGACACCATCACTCTAGATTTCAGTGCAATTTATCTCTTCAAATGTAGC<br>ACCTGAAGTCAGCCCCATACGATATAAGTTGTAATTCTCATGTTAG-<br>TCATGCCCCGCGCCCACCGGAAGGAGCTGACTGGGTTGAAGGCTCTCAAG<br>GGCATCGGTCGAGATCCCGGTGCCTAATGAGTGAGCTAACTTACATTAATT-<br>GCGTT-<br>GCGCTCACTGCCCGCTTTCCAGTCGGGAAACCTGTCGTGCCAGCTGCATTA<br>ATGAATCGGCCAACGCGCGGGGAGAGGCGGTTTGCG-<br>TATTGGGCGCCAGGGTGGTTTTTCTTTTCACCAGTGAGACGGGCAACAGCT<br>GATTGCCCTTCACCGCCTGGCCCTGAGAGAGTTGCAGCAAGCGGTCCAC-<br>GCTGGTTT-<br>GCCCCAGCAGGCGAAAATCCTGTTTGATGGTGGTTAACGGCGGGATATAA<br>CATGAGCTGTCTTCGGTATCGTCGTATCCCACTACCGAGATGTCCGCAC-<br>CAACGCG-<br>CAGCCCGGACTCGGTAATGGCGCGCATTGCGCCCAGCGCCATCTGATCGTT<br>GGCAACCAGCATCGCAGTGGGAACGATGCCCTCATTCAGCATTT-<br>GCATGGTTTGTTGAAAAC-<br>CGGACATGGCACTCCAGTCGCCTTCCCGTTCCGCTATCGGCTGAATTTGATT<br>GCGAGTGAGATATTTATGCCAGCCAGCCAGACGCAGACGCGCCGAGA-<br>CAGAACTTAATGGGCCCGCTAACAGCGCGATTTGCTGGTGACCCAATGCG<br>ACCAGATGCTCCACGCCCAGTCGCGTACCGTCTTCATGGGAGAAAATAA-<br>TACTGTT-<br>GATGGGTGTCTGGTCAGAGACATCAAGAAATAACGCCGGAACATTAGTGC<br>AGGCAGCTTCCACAGCAATGGCATCCTGGTCATCCAGCGGA-<br>TAGTTAATGATCAGCCCAC-<br>TGACGCGTTGCGCGAGAAGATTGTGCACCGCCGCTTTACAGGCTTCGACGC<br>CGCTTCGTTCTACCATCGACACCACCACGCTGGCACCCAGTT-<br>GATCGGCGCGAGAT-<br>TTAATCGCCGCGACAATTTGCGACGGCGCGTGCAGGGCCAGACTGGAGGT<br>GGCAACGCCAATCAGCAACGACTGTTTGCCCGCCAGTTGTTGTGCCAC-<br>GCGGTTGG-<br>GAATGTAATTCAGCTCCGCCATCGCCGCTTCCACTTTTTCCCGCGTTTTCGC<br>AGAAACGTGGCTGGCCTGGTTCACCACGCGGGAAACGGTCTGATAA-<br>GAGACACCGGCAT-<br>ACTCTGCGACATCGTATAACGTTACTGGTTTCACATTCACCACCCTGAATTG<br>ACTCTCTTCCGGGCGCTATCATGCCATACCGCGAAAGGTTTT-<br>GCGCCATTCGATGGTGTCCGGGATCTCGACGCTCTCCCTTATGAAGTCTAA<br>CGCTGCTCTGGGCTAACTGTC

Supplementary Table S5. NIMPLY complex variant sequences used in this study. ACTS Type II N3 and ACTS Type II N7 were colored in red and green, respectively. Linker between the switches was colored in orange. Conserved linker sequence was indicated as gray. Trigger and antisense domain were colored in black. The 5′ and 3′ Overhang domains were colored in blue and purple, respectively. Plasmid sequences of NIMPLY complexes can be constructed by replacing the XOR switch, trigger and antisense region in the example plasmids in Table S4 with the sequences described underneath.

Name	Sequence
**Variant 1 (Used in Figure 2)**	
Switch	ATTGAATATGATAGAAGTTTAGTAGTAGACAATAGAACAGAGGAGATATTGATGACTACTA-<br>AACTAAAACACGCGACTGATTTGAATACACTGCTTCGTTCACGATTCAGAACAGAGGAGATGA<br>ATATGGAACGAAGCAGAAACCTGGCGGCAGCGCAAAAG
Trigger 1	AATTATATATTTTATCACTACTAAACTTCTATCATATTCAATATTTTATTTTATTTTG
Trigger 2	GAAGGGAAAGGGTAGAGAACGAAGCAGTGTATTCAAATCAGTAAAAGAAAAGAGTTGG
Antisense 1	CAAAATAAAATAAAACATTGAATATGATAGAAGTTTAGTAGTAATAAAATATATAATT
Antisense 2	CTAGCTCTTTTCTTTAACTGATTTGAATACACTGCTTCGTTCGCTACCCTTTCCCTTC
**Variant 2**	
Switch	ATTGAATATGATAGAAGTTTAGTAGTACACAATAGAACAGAGGAGATATTGATGACTACTA-<br>AACTAAATAATGGCACTGATTTGAATACACTGCTTCGTTCACGATTCAGAACAGAGGAGATGA<br>ATATGGAACGAAGCAGAAACCTGGCGGCAGCGCAAAAG
Trigger 1	CTTATATTTTACTTTCACTACTAAACTTCTATCATATTCAATAATTTTATTTTATTAC
Trigger 2	GAAAAGTCCTCGGAAAGAACGAAGCAGTGTATTCAAATCAGTAAAAGGGAGAGGATGG
Antisense 1	GTAATAAAATAAAATAATTGAATATGATAGAAGTTTAGTAGTAAAAGTAAAATATAAG
Antisense 2	CCGTCCTCTCCCTTTAACTGATTTGAATACACTGCTTCGTTCCTTCTGAGGACTTTTC
**Variant 3**	
Switch	ATTGAATATGATAGAAGTTTAGTAGTAGACAATAGAACAGAGGAGATATTGATGACTACTA-<br>AACTAAAACCAACGACTGATTTGAATACACTGCTTCGTTCACGATTCAGAACAGAGGAGATGA<br>ATATGGAACGAAGCAGAAACCTGGCGGCAGCGCAAAAG
Trigger 1	CATTTACTTATATTTCACTACTAAACTTCTATCATATTCAATCTTATTATTATTATTC
Trigger 2	GAAGGGAGGGTAGGGAGAACGAAGCAGTGTATTCAAATCAGTATGGGAGAATTTTGGG
Antisense 1	GAATAATAATAATAAAATTGAATATGATAGAAGTTTAGTAGTAAAATATAAGTAAATG
Antisense 2	CCCAAAATTTTCCCGAACTGATTTGAATACACTGCTTCGTTCGCCCTACCTTCCCTTC

Supplementary Table S6. Loop extended switch sequences of NIMPLY complex variant 1 and 2 used in this study. Plasmid sequences of loop extended switch can be constructed by replacing the XOR switch region in the example plasmids in Table S4 with the appropriate switch sequences described underneath. ACTS Type II N3 and ACTS Type II N7 were colored in red and green, respectively. Linker between the switches was colored in orange. Conserved linker sequence was indicated as gray. Extended loop was denoted with blue color.

Name	Ext. length	Sequence
**Variant 1 (Used in Figure 2)**		
Loop ext. 6	6 nt	ATTGAATATGATAGAAGTTTAGTAGTAGACAATAGAACAGAGGAGA-<br>TATTGATGACTACTA-<br>AACTAAAACACGCGACTGATTTGAATACACTGCTTCGTTCACGATTCAA<br>GAAAAGAACAGAGGAGATGAATATGGAACGAAGCAGAAAC-<br>CTGGCGGCAGCGCAAAAG
Loop ext. 9	9 nt	ATTGAATATGATAGAAGTTTAGTAGTAGACAATAGAACAGAGGAGA-<br>TATTGATGACTACTA-<br>AACTAAAACACGCGACTGATTTGAATACACTGCTTCGTTCACGATTCAG<br>AAAGAAAAGAACAGAGGAGATGAATATGGAACGAAGCAGAAAC-<br>CTGGCGGCAGCGCAAAAG

Loop ext. 12	12 nt	ATTGAATATGATAGAAGTTTAGTAGTAGACAATAGAACAGAGGAGA-TATTGATGACTACTA-AACTAAAACACGCGACTGATTTGAATACACTGCTTCGTTCACGATTCAAAAGAAAGAAAAGAACAGAGGAGATGAATATGGAACGAAGCAGAAAC-CTGGCGGCAGCGCAAAAG
**Variant 2**		
Loop ext. 6	6 nt	ATTGAATATGATAGAAGTTTAGTAGTACACAATAGAACAGAGGAGA-TATTGATGACTACTA-AACTAAATAATGGCACTGATTTGAATACACTGCTTCGTTCACGATTCAACGCAAGAACAGAGGAGATGAATATGGAACGAAGCAGAAAC-CTGGCGGCAGCGCAAAAG
Loop ext. 9	9 nt	ATTGAATATGATAGAAGTTTAGTAGTACACAATAGAACAGAGGAGA-TATTGATGACTACTA-AACTAAATAATGGCACTGATTTGAATACACTGCTTCGTTCACGATTCACAAACGCAAGAACAGAGGAGATGAATATGGAACGAAGCAGAAAC-CTGGCGGCAGCGCAAAAG
Loop ext. 12	12 nt	ATTGAATATGATAGAAGTTTAGTAGTACACAATAGAACAGAGGAGA-TATTGATGACTACTA-AACTAAATAATGGCACTGATTTGAATACACTGCTTCGTTCACGATTCAGAACAAACGCAAGAACAGAGGAGATGAATATGGAACGAAGCAGAAAC-CTGGCGGCAGCGCAAAAG

Supplementary Table S7. Trigger and antisense cassette sequences of XOR gate used in this study. Plasmid sequences of trigger and antisense cassettes can be constructed by replacing Table S4 with the cassette sequences described underneath. Colors were identically arranged to Table S4. Lac operator and tet operator were denoted with gray color.

Name	Configuration	Sequence
Trigger cassette	pT7–tetO-Trigger 2–T7term-pT7–lacO-Trigger 1–T7term	TAATACGACTCACTATAGGGTCCCTATCAGTGATAGAGAGAACGAAGCAG-<br>TG-<br>TATTCAAATCAGTAAAAGAAAAGAGTTGGTAGCATAACCCCTTGGGGCCTC<br>TAAACGGGTCTTGAGGGGTTTTTTGCACTTTCGCAAAACTGAGAGCTCAC-<br>GTGTAGGCATTT-<br>GAGAAGCACACGGTCACACTGCTTCCGGTAGTCAATAAACGCTTCTCAAAT<br>GCCTGAGGTTTCAGTAATACGACTCACTATAGGGAATTGTGAGCGGA-<br>TAACAATTCACTACT-<br>AAACTTCTATCATATTCAATATTTTATTTTATTTTGTAGCATTAGCATAACCC<br>CTTGGGGCCTCTAAACGGGTCTTGAGGGGTTTTTTG
Antisense cassette	pT7–lacO-Antisense2–T7term- pT7–tetO-Antisense1–T7term	TAATACGACTCACTATAGGGGAATTGTGAGCGGA-<br>TAACAATTCCTAGCTCTTTTCTTTTACTGATTTGAATACACTGCTTCGTTCTCT<br>ACCCTTTCCCTTCTAGCATAACCCCTTGGGGCCTCTAAACGGGTCTT-<br>GAGGGGTTTTTT-<br>GCTGAAAGGAGGAACTATATCCGGATATCCCGCAAGAGGCCCGGCAGTAC<br>CGGCATAACCAAGCCTATGCCTACAGCATCCAGGGTGACGGTGCCGAG-<br>GATGACGATGAGCG-<br>CATTGTTAGATCCGGATATAGTTCCTCCTTTCAGTAATACGACTCACTATAG<br>GGTCCCTATCAGTGATAGAGACAAAATAAAATAAAATATTGAATATGATA-<br>GAAGTTTAGTAG-<br>TGATAAAATATATAATTTAGCATTAGCATAACCCCTTGGGGCCTCTAAACG<br>GGTCTTGAGGGGTTTTTTG

Supplementary Table S8. Switch sequences of AND gate and NIMPLY gate used in this study. Plasmid sequences of switch RNA can be constructed by replacing Table S4. with the AND switch and NIMPLY switch sequences described underneath. Colors were identically arranged to Table S4. *mCherry* sequence was colored in dark red. Lac operator and tet operator were denoted with gray color.

Name	Configuration	Sequence
AND switch	pT7–AND switch(RBS)–Linker–mCherry–T7term	TAATACGACTCACTATAGGGACTACTATTGATTACAC-<br>GCTTTACTTCGAAATTCATAATGAACAGAGGAGATATGAA<br>ATGCGAAGTAAAGCGAACCTGGCGGCAGCGCAAAA-<br>GATGCGTAAAGTGAGCAAGGGCGAA-<br>GAAGATAACATGGCCATCATCAAGGAGTTCATGCGCTTCA<br>AGGTTCACATGGAGGGCTCCGTGAACGGCCACGAG-<br>TTCGA-<br>GATCGAGGGCGAGGGCGAGGGCCGCCCCTACGAGGGCAC<br>CCAGACCGCCAAGCTGAAGGTGAC-<br>CAAGGGTGGCCCCCTGCCCTTCGCCTGG-<br>GACATCCTGTCCCCTCAGTTCATGTACGGCTCCAAGGCCTA<br>CGTGAAGCACCCCGCCGACATCCCCGACTACTTGAA-<br>GCTGTCCTTCCCCGAGGGCTTCAAGTGGGAGCGCGTGATG<br>AACTTCGAGGACGGCGGCGTGGTGACCGTGACCCAG-<br>GACTCCTCCCTGCAAGACGGCGAG-<br>TTCATCTACAAGGTGAAGCTGCGCGGCACCAACTTCCCCTC<br>CGACGGCCCCGTAATGCAGAAGAAGACTATGGGCTGG-<br>GAGGCCTCCTCCGAGCG-<br>GATGTACCCCGAGGACGGCGCGCTGAAGGGCGAGATCAA

		GCAGAGGCTGAAGCTGAAGGACGGCGGCCACTACGAC-GCTGAGGTCAAGACCAC-CTACAAGGCCAAGAAGCCCGTGCAACTGCCCGGCGCGTACAACGTCAACATCAAGTTGGACATCACCTCCCACAACGAG-GACTACACCATCGTGGAACAG-TACGAACGCGCCGAGGGCCGCCACTCCACCGGCGGCATGGACGAGCTGTACAAGTAATAAAACAG-TCGAGCCCAGCGTGGTTAAACACTCTAG-CATAACCCCTTGGGGCCTCTAAACGGGTCTTGAGGGGTTTTTTG
NIMPLY switch	pT7–NIMPLY switch(RBS)–Linker–mCherry–T7term	TAATACGACTCACTATAGGGGTAATGAATTGTAGGCTT-GTTATAGTTATGAAACAGAGGAGA-CATAACATGAACAAGCCTAACCTGGCGGCAGCGCAAAAGATGCGTAAAGTGAGCAAGGGCGAAGAAGA-TAACATGGCCATCATCAAGGAG-TTCATGCGCTTCAAGGTTCACATGGAGGGCTCCGTGAACGGCCACGAGTTCGA-GATCGAGGGCGAGGGCGAGGGCCGCCCCTACGAGGG-CACCCAGAC-CGCCAAGCTGAAGGTGACCAAGGGTGGCCCCCTGCCCTTCGCCTGGGACATCCTGTCCCCTCAGTTCATGTAC-GGCTCCAAGGCCTACGTGAA-GCACCCCGCCGACATCCCCGACTACTTGAAGCTGTCCTTCCCCGAGGGCTTCAAGTGGGAGCGCGTGATGAACTTCGAG-GACGGCGGCGTGGTGAC-CGTGACCCAGGACTCCTCCCTGCAAGACGGCGAGTTCATCTACAAGGTGAAGCTGCGCGGCACCAACTTCCCCTCCGAC-GGCCCCGTAATGCAGAAGAAGAC-TATGGGCTGGGAGGCCTCCTCCGAGCGGATGTACCCCGAGGACGGCGCGCTGAAGGGCGAGATCAAGCAGAGGCTGAA-GCTGAAGGACGGCGGCCACTAC-GACGCTGAGGTCAAGACCACCTACAAGGCCAAGAAGCCCGTGCAACTGCCCGGCGCGTACAACGTCAACATCAAGTT-GGACATCACCTCCCACAACGAG-GACTACACCATCGTGGAACAGTACGAACGCGCCGAGGGCCGCCACTCCACCGGCGGCATGGACGAGCTGTACAAGTAA-TAAAACAG-TCGAGCCCAGCGTGGTTAAACACTCTAGCATAACCCCTTGGGGCCTCTAAACGGGTCTTGAGGGGTTTTTTG

Supplementary Table S9. Trigger and antisense cassette sequences of AND gate and NIMPLY gate used in this study. Plasmid sequences of trigger and antisense cassette can be constructed by replacing Table S4 with the cassette sequences described underneath. Colors were identically arranged to Table S4. Lac operator and tet operator were denoted with gray color.

Name	Configuration	Sequence
**AND gate**		
Trigger cassette	pT7–tetO-Trigger 2–T7term-pT7–lacO-Trigger 1–T7term-	TAATACGACTCACTATAGGGTCCCTATCAGTGATAGAGAGAACGAAGCAGTG-TATTCAAATCAGTAAAAGAAAAGAGTTGGTAGCATAACCCCTTGGGGCCTCTAAACGGGTCTTGAGGGGTTTTTTGCACTTTCGCAAAACTGAGAGCTCACGTG-TAGGCATTTGA-GAAGCACACGGTCACACTGCTTCCGGTAGTCAATAAACGCTTCTCAAATGCCT

	pT7–lacO-AND trigger–T7term	GAGGTTTCAGTAATACGACTCACTATAGGGAATTGTGAGCGGA-TAACAATTCACTACTA-AACTTCTATCATATTCAATATTTTATTTTATTTTGTAGCATTAGCATAACCCCTTGGGGCCTCTAAACGGGTCTTGAGGGGTTTTTTGGCGCGGCGTAGACACCAGA-CAAACGGC-TATCACGGTAAACCAGCAATAGACATAAGCGGCTATTTAACGACCCTGCCCTGAACCGCGCTAATACGACTCACTATAGGGGAATTGTGAGCGGA-TAACAATTCCCAAGCACAC-GAGCTTGGATACAATCTTCACTCCATCTCCATCCATAATCAATAGTAGTCAATAGCATAACCCCTTGGGGCCTCTAAACGGGTCTTGAGGGGTTTTTTG
Antisense cassette	pT7–lacO-Antisense2–T7term-pT7–tetO-Antisense1–T7term-pT7–tetO-AND antisense–T7term	TAATACGACTCACTATAGGGGAATTGTGAGCGGA-TAACAATTCCTAGCTCTTTTCTTTTACTGATTTGAATACACTGCTTCGTTCTCTACCCTTTCCCTTCTAGCATAACCCCTTGGGGCCTCTAAACGGGTCTT-GAGGGGTTTTTT-GCTGAAAGGAGGAACTATATCCGGATATCCCGCAAGAGGCCCGGCAGTACCGGCATAACCAAGCCTATGCCTACAGCATCCAGGGTGACGGTGCCGAGGATGAC-GATGAGCG-CATTGTTAGATCCGGATATAGTTCCTCCTTTCAGTAATACGACTCACTATAGGGTCCCTATCAGTGATAGAGACAAAATAAAATAAAATATTGAATATGATA-GAAGTTTAGTAG-TGATAAAATATATAATTTAGCATTAGCATAACCCCTTGGGGCCTCTAAACGGGTCTTGAGGGGTTTTTTGGCGCAACGAAAGCCAGATTTTCATACAC-GGTGCCTGACTGCGTTAG-CAATTTAACTGTGATAAACTACCGCATTAAAGCTTATCGATGATAAGCTGTCAAACATGAGAATTCTTGAAGGCCGTAATACGACTCACTATAGGGTCCCTATCAG-TGATAGA-GAACGAGGTATGGTCCTCGTAAACGAAGTAAAGCGTGAGCATGGAGATGGAGTGAAGATTGGGATAGCATAACCCCTTGGGGCCTCTAAACGGGTCTT-GAGGGGTTTTTTG
**NIMPLY gate**		
Trigger cassette	pT7–tetO-Trigger 2–T7term-pT7–lacO-Trigger 1–T7term-pT7–tetO-NIMPLY trigger–T7term	TAATACGACTCACTATAGGGTCCCTATCAGTGATAGAGAGAACGAAGCAGTG-TATTCAAATCAGTAAAAGAAAAGAGTTGGTAGCATAACCCCTTGGGGCCTCTAAACGGGTCTTGAGGGGTTTTTTGCACTTTCGCAAAACTGAGAGCTCACGTG-TAGGCATTTGA-GAAGCACACGGTCACACTGCTTCCGGTAGTCAATAAACGCTTCTCAAATGCCTGAGGTTTCAGTAATACGACTCACTATAGGGAATTGTGAGCGGA-TAACAATTCACTACTA-AACTTCTATCATATTCAATATTTTATTTTATTTTGTAGCATTAGCATAACCCCTTGGGGCCTCTAAACGGGTCTTGAGGGGTTTTTTGGCGCGGCGTAGACACCAGA-CAAACGGC-TATCACGGTAAACCAGCAATAGACATAAGCGGCTATTTAACGACCCTGCCCTGAACCGCGCTAATACGACTCACTATAGGGTCCCTATCAGTGATAGAGAAC-CGTGGACCG-CATGAGGTCCACGGTAAACATAACTATAACAAGCCTACAATTCATTACTTTACTAGCGCCATCAACTAGCATAACCCCTTGGGGCCTCTAAACGGGTCTT-GAGGGGTTTTTTG
Antisense cassette	pT7–lacO-Antisense2–T7term-pT7–tetO-Antisense1–T7term-	TAATACGACTCACTATAGGGGAATTGTGAGCGGA-TAACAATTCCTAGCTCTTTTCTTTTACTGATTTGAATACACTGCTTCGTTCTCTACCCTTTCCCTTCTAGCATAACCCCTTGGGGCCTCTAAACGGGTCTT-GAGGGGTTTTTT-GCTGAAAGGAGGAACTATATCCGGATATCCCGCAAGAGGCCCGGCAGTACCG

	pT7–lacO-NIMPLY antisense–T7term	GCATAACCAAGCCTATGCCTACAGCATCCAGGGTGACGGTGCCGAGGATGAC-GATGAGCG-CATTGTTAGATCCGGATATAGTTCCTCCTTTCAGTAATACGACTCACTATAGGGTCCCTATCAGTGATAGAGACAAAATAAAATAAAATATTGAATATGATA-GAAGTTTAGTAG-TGATAAAATATATAATTTAGCATTAGCATAACCCCTTGGGGCCTCTAAACGGGTCTTGAGGGGTTTTTTGGCGCAACGAAAGCCAGATTTCATACAC-GGTGCCTGACTGCGTTAG-CAATTTAACTGTGATAAACTACCGCATTAAAGCTTATCGATGATAAGCTGTCAAACATGAGAATTCTTGAAGGCGCTAATACGACTCACTATAGGGAATT-GTGAGCGGA-TAACAATTCCACGATGCGCATTTACGCATCGTGGATGGATGGCGCTAGTAAAGTAATGAATTGTAGGCTTGTTATAGTTATGAAGTAGCATAACCCCTT-GGGGCCTCTAAACGGGTCTTGAGGGGTTTTTTG

Supplementary Table S10. Three-way junction (3WJ) repressor and small transcription activating RNA (STAR) sequences used in this study. 3WJrep_N19 switch and trigger were used in figure S16 [1]. AD1.S5, T181.S7 and cognate STAR triggers were used in figure S18 [2]. Colors were identically arranged to Table S5. In addition, hairpin structure attached ahead of trigger was denoted in green. Plasmid sequences of 3WJ repressor and STAR can be constructed by replacing the AND/NIMPLY switch, trigger and antisense region in the example plasmids in Table S4 with the sequences described underneath. To note, pLlacO-1 promoter was used for 3WJ trigger transcription and STAR switch transcription.

Name	Sequence
**3WJ repressor**	
3WJrep_N19 switch	ACTAATCAGATCTACTTGTTATAGTTATGAACAGAGGAGA-CATAACATGAACAAGCAC-CTAACAAGACTAATCAACCTGGCGGCAGCGCAAAAG
3WJrep_N19 trigger-5′+3′OH	GTGAGCGCAAAGGCTCACTTAAGATGTAAATGTAAACTTGTTAGGTGCG-TAGATCTGATTAGTGTGACCGAGAATGAA
3WJrep_N19 antisense-5′+3′OH	GGTCCCTGCGATGGGACCCTATTCATTCTCGGTCACACTAATCAGATCTAC-GCACCTAACAAGTTTACATTTACATCT
3WJrep_N19 trigger-5′OH	GAGCGCGATTCAGCGCTCATCGAATGTATATGTAAACTTGTTAGGTGCG-TAGATCTGATTAGT
3WJrep_N19 antisense-3′OH	CCGGACTAACACGTCCGGACAGCACCTAACAAGTTTACATATACATTC
**STAR AD1**	
AD1.S5 switch	AGTTTTTACAGTGAATTGTTTTAATTAGTTGTATAAATGTTGGAG-CAGCGGGGAATGTATA-CAGTTCATGTATATATTCCCCGCTTTTTTTTTAACCTGGCGGCAGCGCAAAAGAGAGGAGACAGAG
AD1 Trigger-3′OH	TGAACTGTATACATTCCCCGCTGCTCCAACATTTATA-CAACTAATTAAAACAATTCACTGTAAAAACTTCCATCCTATATCCC
AD1 Antisense-5′OH	GCGCAGACTAACCTGCGCAATGGGATATAGGATGGAAGTTTTTACAG-TGAATTGTTTTAATTAGTTGTATAAATGTTGGAGCA
AD1 Trigger-3′OH_6nt	TGAACTGTATACATTCCCCGCTGCTCCAACATTTATA-CAACTAATTAAAACAATTCACTGTAAAAACTTATCCATCATCTATC
AD1 Antisense-5′OH_6nt	GTGCACATGTAAGTGCACAATGATAGATGATGGATAAGTTTTTACAG-TGAATTGTTTTAATTAGTTGTATAAATGTTGGAGCAGCGGGG
**STAR T181**	

T181.S7 switch	ACATAAAGATATATATTTGGGTGAGCGATTCCTTAAACGAAATTGAGAT-TAAGGAG-TCGCTCTTTTTTTTTTAACCTGGCGGCAGCGCAAAAGAGAGGAGACAGAG
T181 Trigger-3′OH	TAAGGAATCGCTCACCCAAATATATATCTTTATGTCTACCCGACAATTACT
T181 Antisense-5′OH	CCCATCACAGGCGATGGGAATAGTAATTGTCGGGTAGACATAAA-GATATATATTTGGGT
T181 Trigger-3′OH_6nt	TAAGGAATCGCTCACCCAAATATATATCTTTATGTCTCCAATTCATTACTC
T181 Antisense-5′OH_6nt	GCCGACGACCGAGTCGGCGATGAGTAATGAATTGGAGACATAAA-GATATATATTTGGGTGAGCGA

Supplementary Table S11. Other accessory sequences used in this study. Accessory sequences used for constructing plasmids are indicated here.

Name	**Sequence**
RBS	AGAGGAGA
Linker	AACCTGGCGGCAGCGCAAAAG
Lac operator	AATTGTGAGCGGATAACAATTC
Tet operator	TCCCTATCAGTGATAGAGA
T7term	TAGCATAACCCCTTGGGGCCTCTAAACGGGTCTT-GAGGGGTTTTTTG
GFPmut3b_ASV	ATGCGTAAAGGAGAAGAACTTTTCACTGGAGTT-GTCCCAATTCTTGTTGAATT-AGATGGTGATGTTAATGGGCACAAATTTTCTGTCAGTGGAGAGGGTGAAGGTGATGCAACAT-ACGGAAAACTTACCCTTAAATTTATTTGCACTACTGGAAAAC-TACCTGTTCCGTGGCCAACACTTGTCACTACTTTCGGTTATGGTGTTCAATGCTTTGCGAGATACCCAGATCACATGAAACAG-CATGACTTTTTCAAGAG-TGCCATGCCCGAAGGTTACGTACAGGAAAGAACTATATTTTTCAAAGATGACGGGAACTACAAGACACGTGCTGAAGTCAAGTTT-GAAGGTGATACCCTTGTTAA-TAGAATCGAGTTAAAAGGTATTGATTTTAAAGAAGATGGAAACATTCTTGGACACAAATTGGAATACAAC-TATAACTCACACAATGTATACATCATGGCAGA-CAAACAAAAGAATGGAATCAAAGTTAACTTCAAAATTAGACACAACATTGAAGATGGAAGCGTTCAACTAGCAGAC-CATTATCAACAAAA-TACTCCGATTGGCGATGGCCCTGTCCTTTTACCAGACAACCATTACCTGTCCACACAATCTGCCCTTTCGAAAGATCCCAAC-GAAAAGAGAGAC-CACATGGTCCTTCTTGAGTTTGTAACCGCTGCTGGGATTACACATGGCATGGATGAACTATACAAAAGGCCTGCAGCAAACGAC-GAAAACTACGCTGCATCAGTTTAATAA

mCherry	ATGGGCAGCAGCCATCATCATCATCATCACAG-<br>CAGCGGCCTGGTGCCGCGCGG-<br>CAGCCATATGCGTAAAATGGTGAGCAAGGGCGAAGAAGATAA<br>CATGGCCATCATCAAGGAG-<br>TTCATGCGCTTCAAGGTTCACATGGAGGGCTCCGTGAAC-<br>GGCCACGAGTTCGAGATCGAGGGCGAGGGCGAGGGCCGCCCC<br>TACGAGGGCACCCAGACCGCCAAGCTGAAGGTGAC-<br>CAAGGGTGGCCCCCTGCCCTTCGCCTGGGACATCCTGTCCCCT<br>CAGTTCATGTACGGCTCCAAGGCCTACGTGAA-<br>GCACCCCGCCGACATCCCCGACTACTTGAA-<br>GCTGTCCTTCCCCGAGGGCTTCAAGTGGGAGCGCGTGATGAAC<br>TTCGAGGACGGCGGCGTGGTGACCGTGACCCAG-<br>GACTCCTCCCTGCAAGACGGCGAG-<br>TTCATCTACAAGGTGAAGCTGCGCGGCACCAACTTCCCCTCCG<br>ACGGCCCCGTAATGCAGAAGAAGACTATGGGCTGG-<br>GAGGCCTCCTCCGAGCG-<br>GATGTACCCCGAGGACGGCGCGCTGAAGGGCGAGATCAAGCA<br>GAGGCTGAAGCTGAAGGACGGCGGCCACTACGAC-<br>GCTGAGGTCAAGACCAC-<br>CTACAAGGCCAAGAAGCCCGTGCAACTGCCCGGCGCGTACAA<br>CGTCAACATCAAGTTGGACATCACCTCCCACAACGAGGAC-<br>TACACCATCGTGGAACAGTAC-<br>GAACGCGCCGAGGGCCGCCACTCCACCGGCGGCATGGACGAG<br>CTGTACAAGTAA

Supplementary Figures

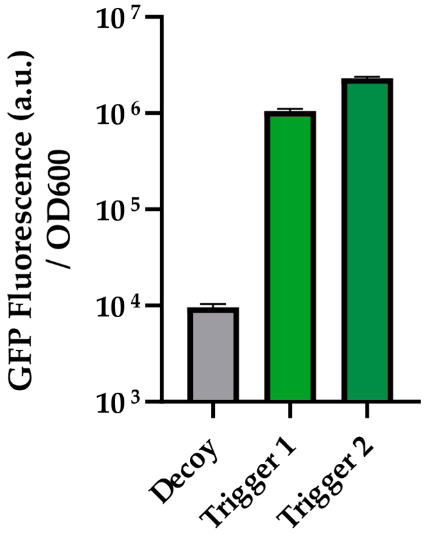

Supplementary Figure S1. OR gate used in this study. ACTS Type II N3 and ACTS Type II N7 from the previous research [3] was adopted to 2-input OR gate with 9 nucleotide linker sequence. T7 RNA polymerase was induced by 1 mM IPTG in *E. coli* BL21 DE3 strain. GFP fluorescence was measured on the microplate reader (error bars indicate ± SD from three biological replicates).

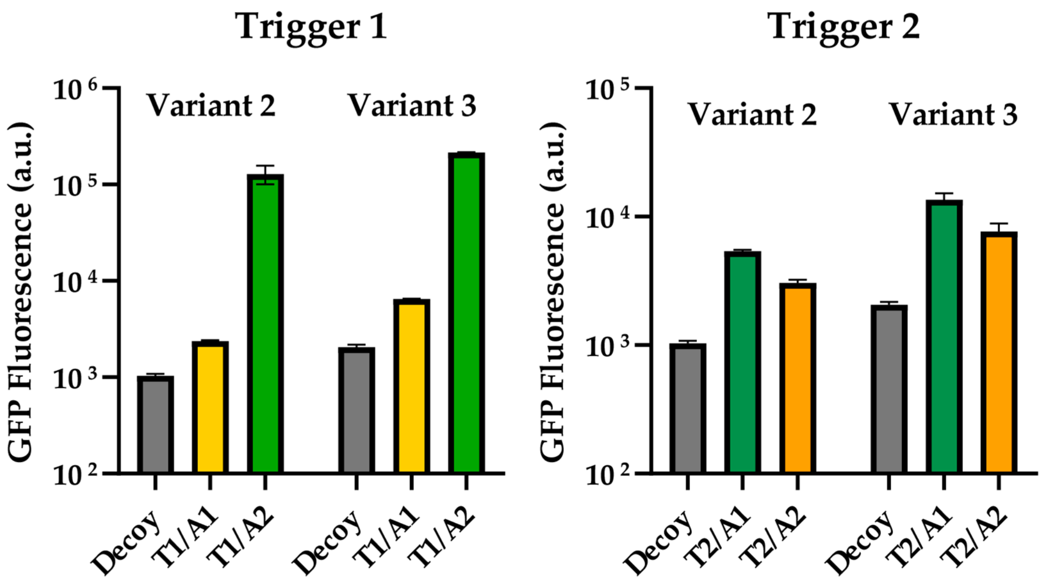

Supplementary Figure S2. Performance of NIMPLY complex of variant 2 and 3. Variant 2 and 3 was built with same design scheme of variant 1 that used in Figure 2. T7 RNA polymerase was induced by 1 mM IPTG in *E. coli* BL21 DE3 strain. GFP fluorescence was measured on the flow cytometry (error bars indicate ±SD from three biological replicates). Cellular autofluorescence was subtracted in all cases. Autofluorescence level was measured from cells not bearing a GFP-expressing plasmid.

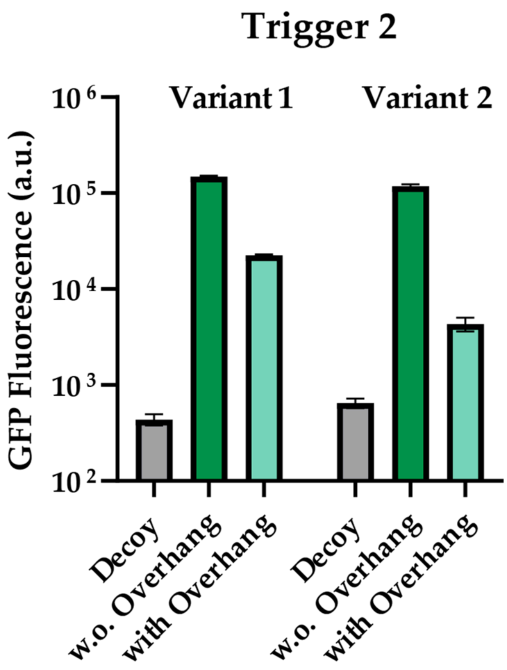

Supplementary Figure S3. Weak activation of trigger RNA with overhang sequence. Variant 1 was the NIMPLY complex used in Figure 2. Overhang comprised of both 5′ and 3′ overhang sequence. T7 RNA polymerase was induced by 1 mM IPTG in *E. coli* BL21 DE3 strain. GFP fluorescence was measured on the flow cytometry (error bars indicate ± SD from three biological replicates). Cellular autofluorescence was subtracted in all cases. Autofluorescence level was measured from cells not bearing a GFP-expressing plasmid.

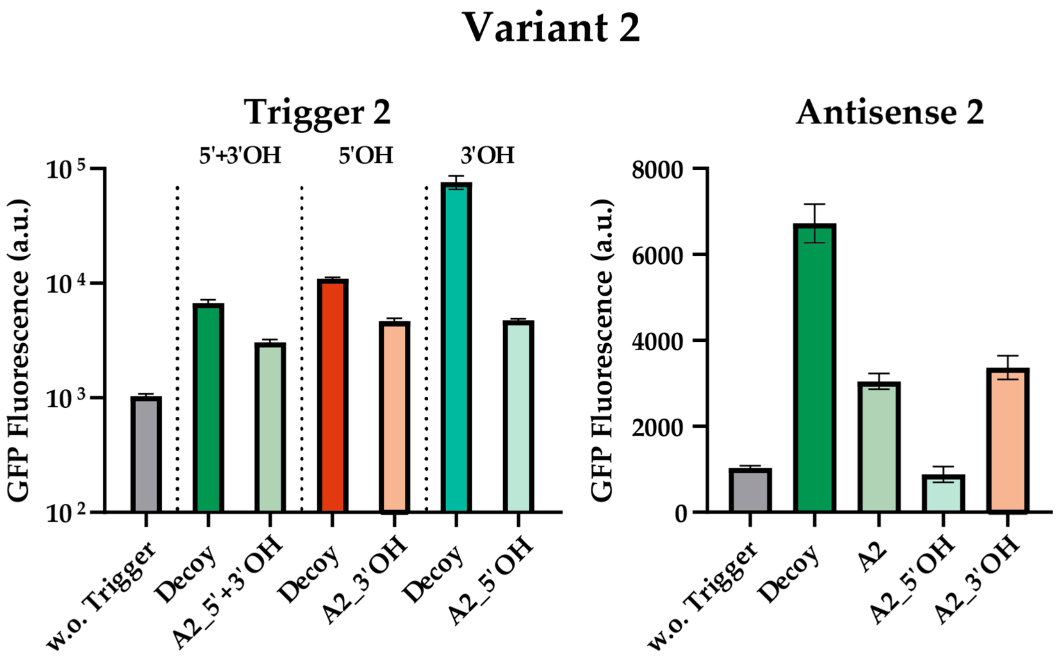

Supplementary Figure S4. Effect of overhang sequence on variant 2. A2 denoted antisense RNA that responds to trigger 2. Existing overhang sequence was denoted as 5′ or 3′ OH. Without Trigger indicated that only NIMPLY complex was treated in the system. T7 RNA polymerase was induced by 1 mM IPTG in *E. coli* BL21 DE3 strain. GFP fluorescence was measured on the flow cytometry (error bars indicate ± SD from three biological replicates). Cellular autofluorescence was subtracted in all cases. Autofluorescence level was measured from cells not bearing a GFP-expressing plasmid.

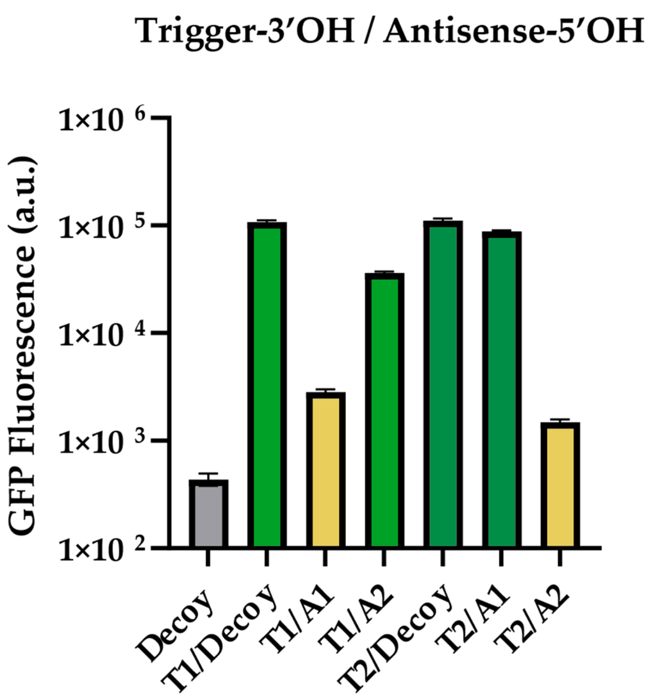

Supplementary Figure S5. Performance of NIMPLY complex with overhang-deleted constructs. Experiment was performed using variant 1. Trigger RNA 1 and 2 only consisted of 3′ overhang sequence. Antisense RNA 1 and 2 comprised of 5′ overhang sequence. Significant decrease in T1/Decoy to T1/A2 indicated cross-reactivity. T7 RNA polymerase was induced by 1 mM IPTG in *E. coli* BL21 DE3 strain. GFP fluorescence was measured on the flow cytometry (error bars indicate ±SD from three biological replicates). Cellular autofluorescence was subtracted in all cases. Autofluorescence level was measured from cells not bearing a GFP-expressing plasmid.

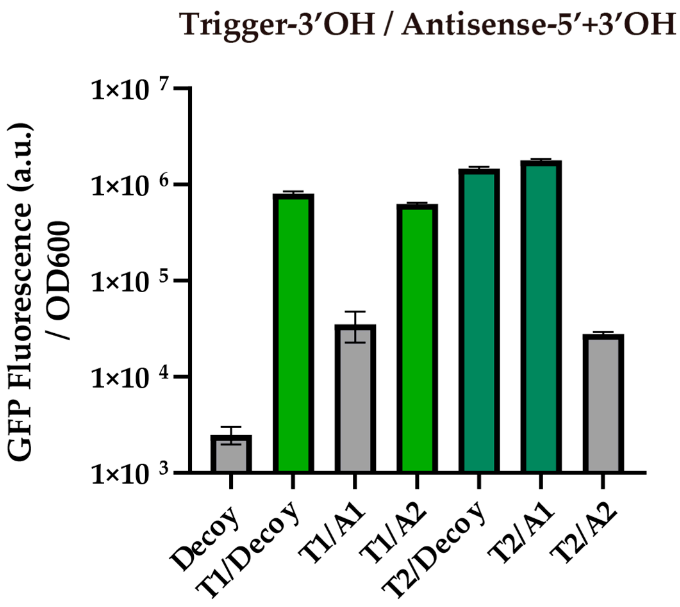

Supplementary Figure S6. Performance of NIMPLY complex with overhang-deleted trigger RNAs. Experiment was performed using variant 1. Trigger RNA 1 and 2 only consisted of 3′ overhang sequence. Antisense RNA 1 and 2 comprised of both 5′ and 3′ overhang sequence. No significant decrease in T1/Decoy to T1/A2 that observed in Figure S5 was not detected with full-length antisense RNA. Fold repression evaluated with ON-to-Repressed state of reporter expression was 17.8-fold for T1 and 64.1-fold for T2. T7 RNA polymerase was induced by 1 mM IPTG in *E. coli* BL21 DE3 strain. GFP fluorescence was measured on the microplate reader (error bars indicate ±SD from three biological replicates).

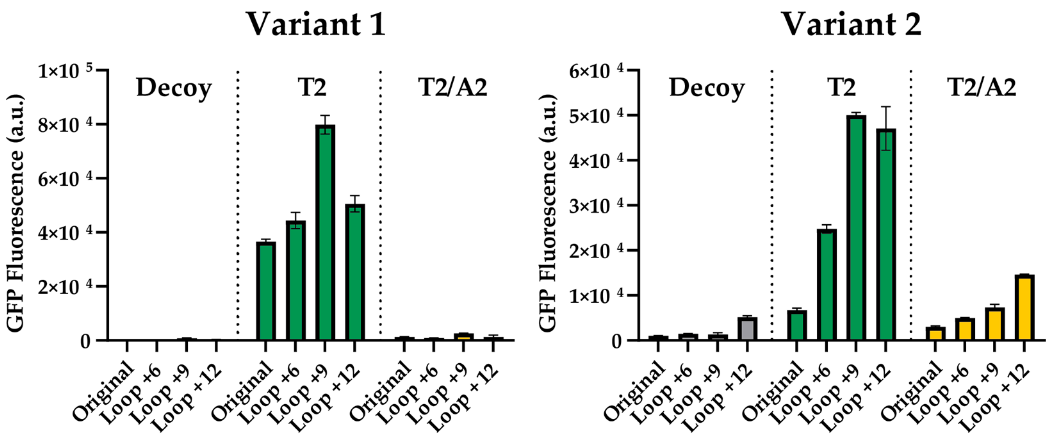

Supplementary Figure S7. Effect of loop size on trigger activity on variant 1 and 2. Variant 1 indicated the NIMPLY complex used in Figure 2. Enlargement of hairpin loop of the NIMPLY gate switch 2 was denoted as Loop +6/9/12. 6/9/12 nucleotides were inserted to the position between the stem and loop interval. Increment of the hairpin loop drastically upregulated the ON level expression on both variant 1 and 2, but leaky expression was also detected on variant 2. T7 RNA polymerase was induced by 1 mM IPTG in *E. coli* BL21 DE3 strain. GFP fluorescence was measured on the flow cytometry (error bars indicate ± SD from three biological replicates). Cellular autofluorescence was subtracted in all cases. Autofluorescence level was measured from cells not bearing a GFP-expressing plasmid.

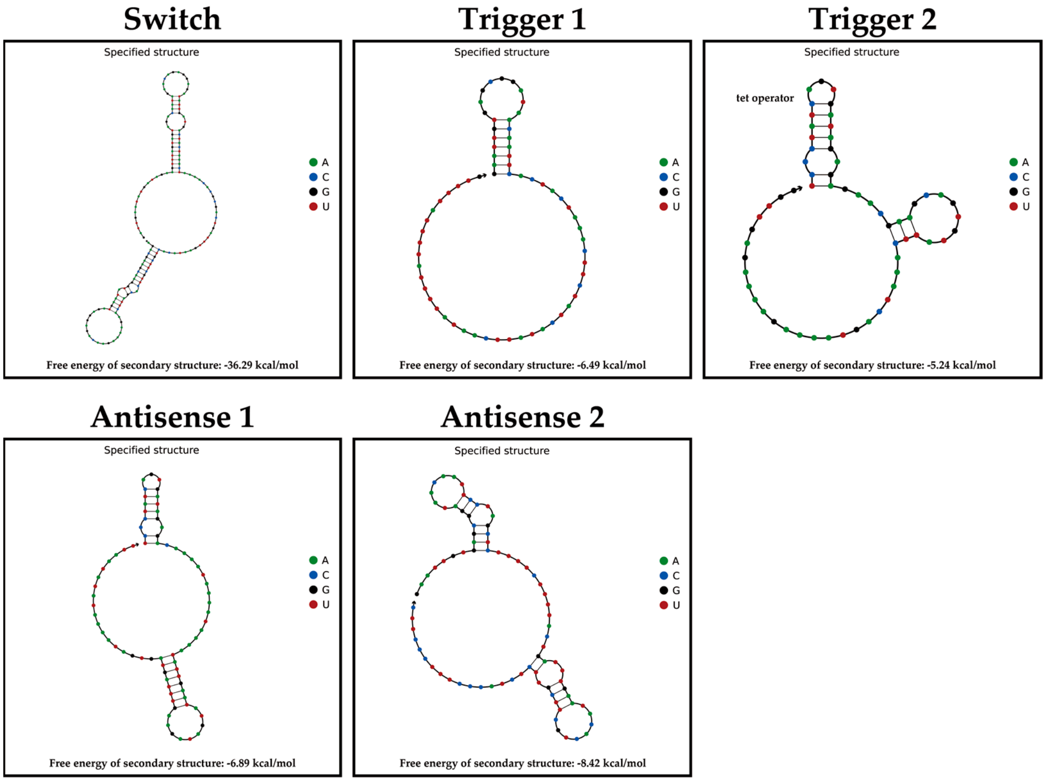

Supplementary Figure S8. Predicted minimum free energy (MFE) structure for XOR gate components. Lac and tet operators were attached to the 5′ region of appropriate trigger and antisense RNAs. NUPACK was used to calculate each MFE structures.

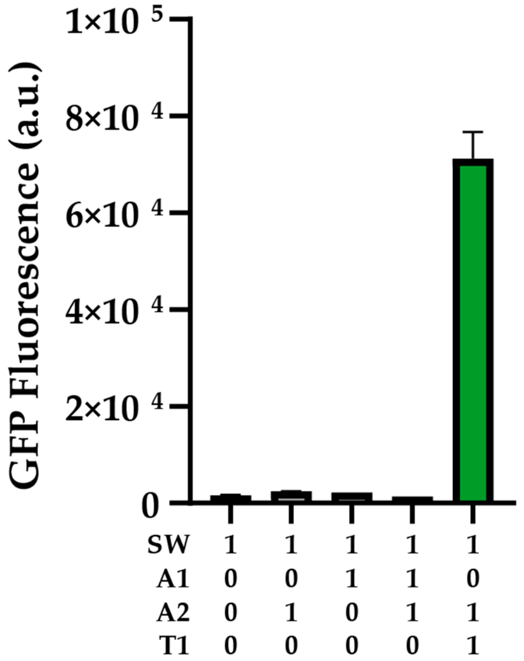

Supplementary Figure S9. Cross-reactivity between switch RNA and antisense cassette. Combinations of input RNA was displayed in table. No significant increase in reporter expression was observed with either inducer treated. T7 RNA polymerase was induced with the pretreatment of 1 % (w/w) arabinose in *E. coli* BL21 AI strain. Antisense RNA was induced by 1 mM IPTG and 200 ng/mL aTc. GFP fluorescence was measured on flow cytometry (error bars indicate ±SD from three biological replicates). Cellular autofluorescence was subtracted in all cases. Autofluorescence level was measured from cells not bearing a GFP-expressing plasmid.

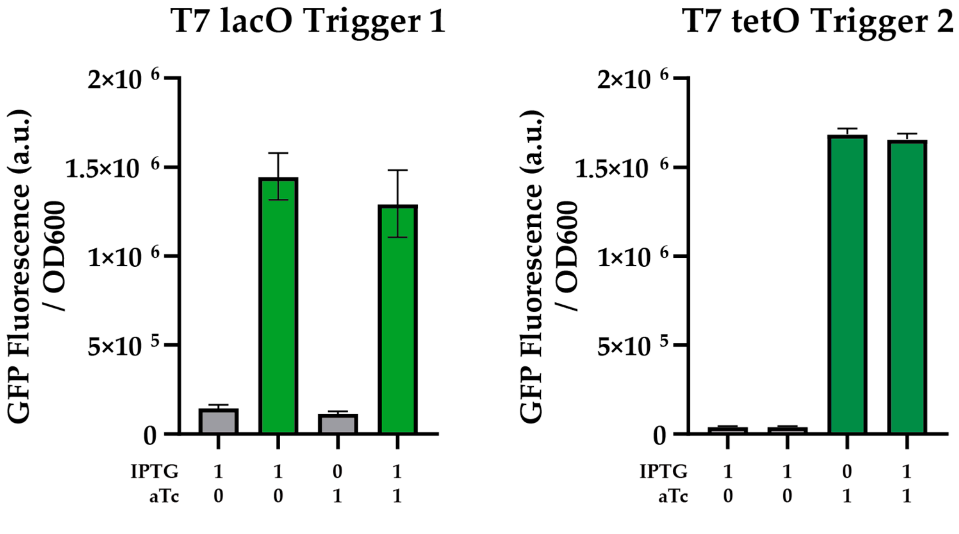

Supplementary Figure S10. Chemically inducible trigger RNA 1 and 2. Trigger RNA 1 and 2 with lac operator and tet operator, respectively, was expressed with OR gate of NIMPLY complex. Notably, both triggers were exhibited fairly high ON level expression when appropriate inducer was treated. However, little leak transcription was detected on T7 promoter with lac operator in trigger 1. Presence of IPTG and aTc was displayed in table. T7 RNA polymerase was induced with the pretreatment of 1 % (w/w) arabinose in *E. coli* BL21 AI strain. Trigger RNA was induced by 1 mM IPTG and 200 ng/mL aTc. GFP fluorescence was measured on flow cytometry (error bars indicate ±SD from three biological replicates). Cellular autofluorescence was subtracted in all cases. Autofluorescence level was measured from cells not bearing a GFP-expressing plasmid.

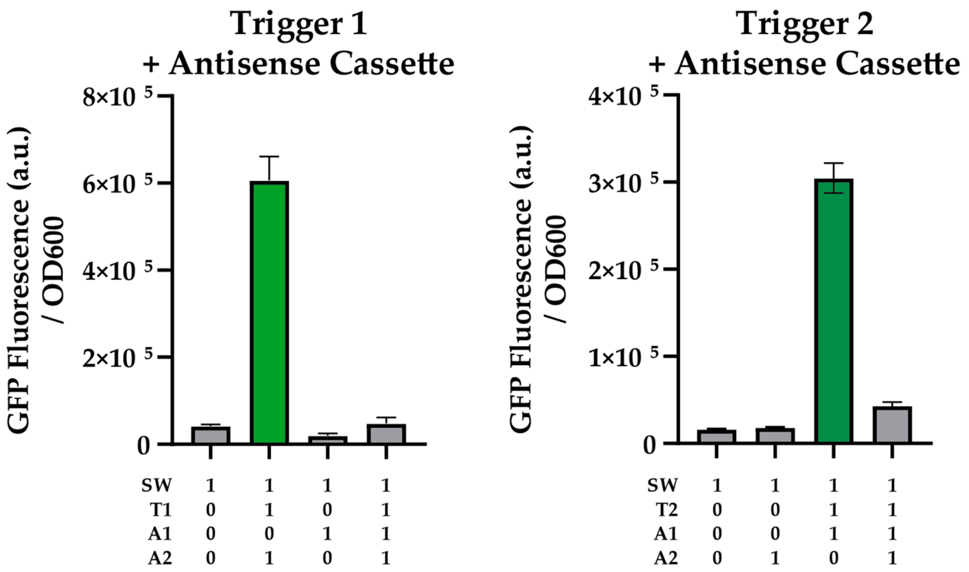

Supplementary Figure S11. Investigation of chemically inducible antisense cassette on trigger 1 and 2. Trigger RNA 1 and 2 with lac operator and tet operator, respectively, was expressed with OR gate and antisense cassette of NIMPLY complex. Antisense cassette was composed of antisense RNA 1 and 2 with tet operator and lac operator, respectively. Both triggers were exhibited ON level expression when appropriate inducer was treated. Notably, repressed state was observed when matching antisense RNA was expressed. Combinations of input RNA was displayed in table. T7 RNA polymerase was induced with the pretreatment of 1 % (w/w) arabinose in *E. coli* BL21 AI strain. Trigger and antisense RNA were induced by 1 mM IPTG and 200 ng/mL aTc. GFP fluorescence was measured on flow cytometry (error bars indicate ±SD from three biological replicates). Cellular autofluorescence was subtracted in all cases. Autofluorescence level was measured from cells not bearing a GFP-expressing plasmid.

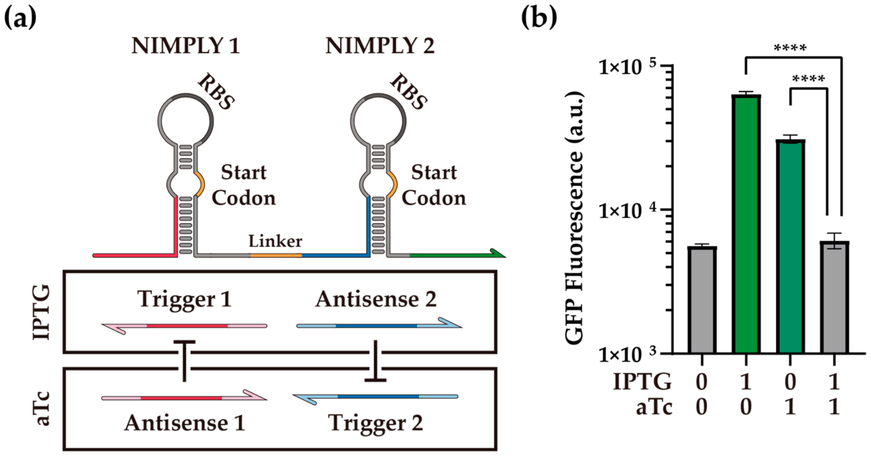

Supplementary Figure S12. Toehold switch based XOR gate with full-length trigger RNAs. (a) Schematics of XOR gate. Lac operator was arranged ahead of T1 and A2, and Tet operator was arranged in front of T2 and A1. (b) Performance of XOR gate with Full-length trigger. Presence of IPTG and aTc was displayed in table. Statistical analysis was performed for comparing each state of XOR gate. T7 RNA polymerase was induced with the pretreatment of 1 % (w/w) arabinose in *E. coli* BL21 AI strain. RNAs of XOR gate were induced by 1 mM IPTG and 200 ng/mL aTc. GFP fluorescence was measured on flow cytometry. Cellular autofluorescence was subtracted in all cases. Autofluorescence level was measured from cells not bearing a GFP-expressing plasmid. (two-tailed Student's t-test; **** $p < 0.0001$; Error bars indicate ± SD from three biological replicates)

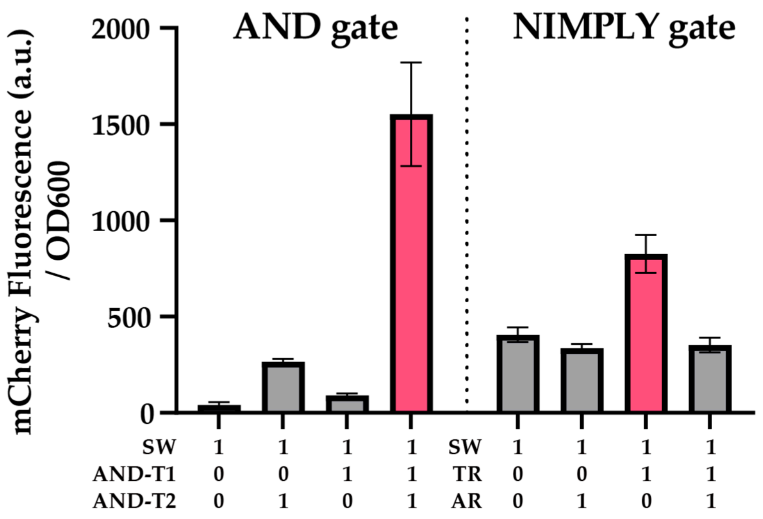

Supplementary Figure S13. Chemically inducible AND gate and NIMPLY gate for half adder and half subtractor. AND gate was derived from DNF circuit of previous research and another de novo NIMPLY gate was constructed with TS_gen1_N001 [3]. Then, lac operator and tet operator were attached to the input signals of AND gate and NIMPLY gate. Combinations of input RNA was displayed in table. T7 RNA polymerase was induced with the pretreatment of 1 % (w/w) arabinose in *E. coli* BL21 AI strain. AND triggers, NIMPLY trigger and NIMPLY antisense RNA were induced by 1 mM IPTG and 200 ng/mL aTc. GFP fluorescence was measured on flow cytometry (error bars indicate ±SD from three biological replicates). Cellular autofluorescence was subtracted in all cases. Autofluorescence level was measured from cells not bearing a GFP-expressing plasmid.

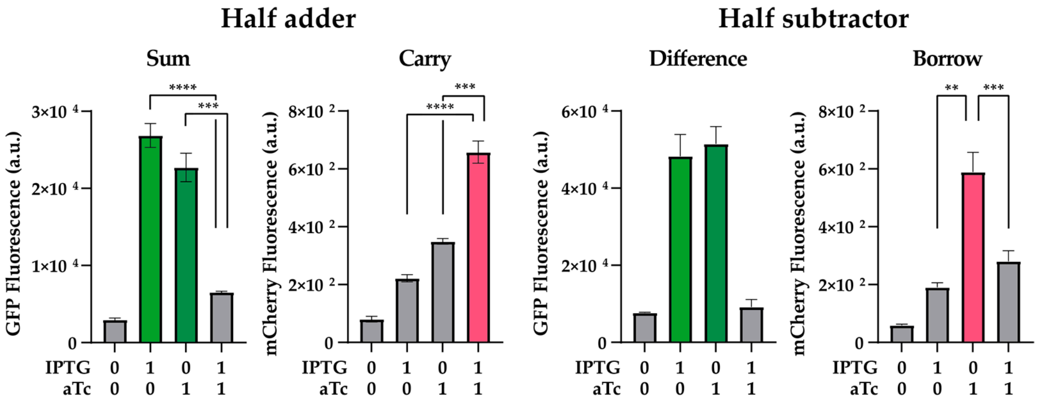

Supplementary Figure S14. Chemically inducible half adder and half subtractor. Performances of half adder and half subtractor were displayed in bar chart. Presence of IPTG and aTc was displayed in table. Statistical analysis was performed for comparing each state of binary calculators. T7 RNA polymerase was induced with the pretreatment of 1 % (w/w) arabinose in *E. coli* BL21 AI strain. RNAs of half adder and half subtractor were induced by 1 mM IPTG and 200 ng/mL aTc. GFP fluorescence was measured on flow cytometry. Cellular autofluorescence was subtracted in all cases. Autofluorescence level was measured from cells not bearing a GFP-expressing plasmid. (two-tailed Student's t-test; ** $p < 0.01$; *** $p < 0.001$; **** $p < 0.0001$; Error bars indicate ± SD from three biological replicates)

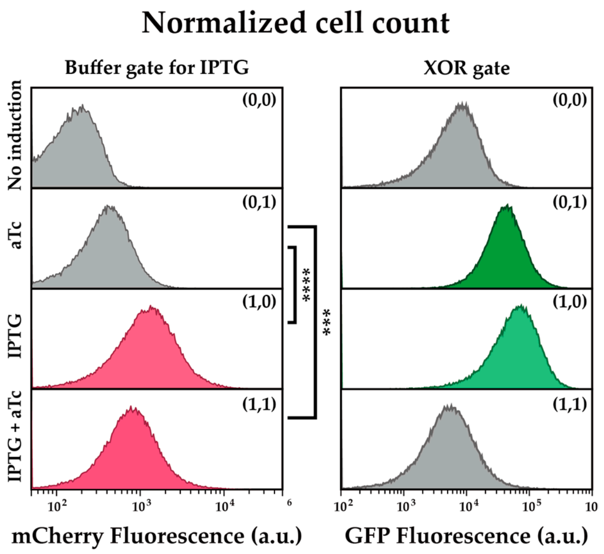

Supplementary Figure S15. Flow cytometry GFP and mCherry fluorescence histograms for Feynman gate. Presence of IPTG and aTc was displayed on bracket. T7 RNA polymerase was induced with the pretreatment of 1 % (w/w) arabinose in *E. coli* BL21 AI strain. AND triggers, NIMPLY trigger and NIMPLY antisense RNA were induced by 1 mM IPTG and 200 ng/mL aTc. GFP fluorescence was measured on flow cytometry. (two-tailed Student's t-test; *** $p < 0.001$; **** $p < 0.0001$)

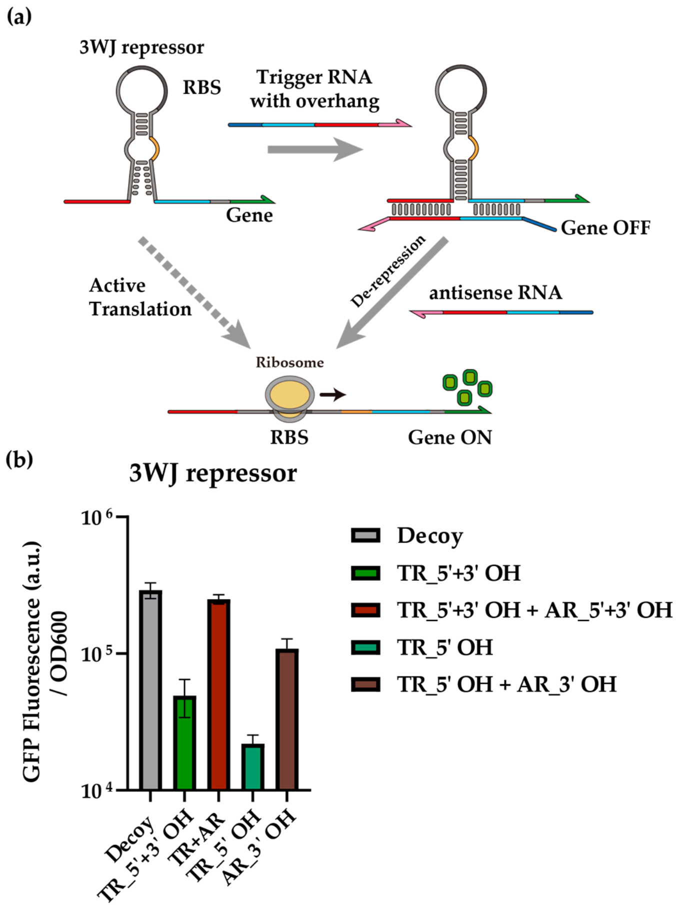

Supplementary Figure S16. De-repressing activity of antisense RNA in 3WJ repressor. 3WJ repressor was derived from previous research [1]. Trigger RNA and antisense RNA composed of single or dual overhang was tested. Significant de-repressing activity that almost recovers the ON state

of 3WJ repressor was observed. T7 RNA polymerase was induced by 1 mM IPTG in *E. coli* BL21 DE3 strain. GFP fluorescence was measured on the microplate reader (error bars indicate ±SD from three biological replicates).

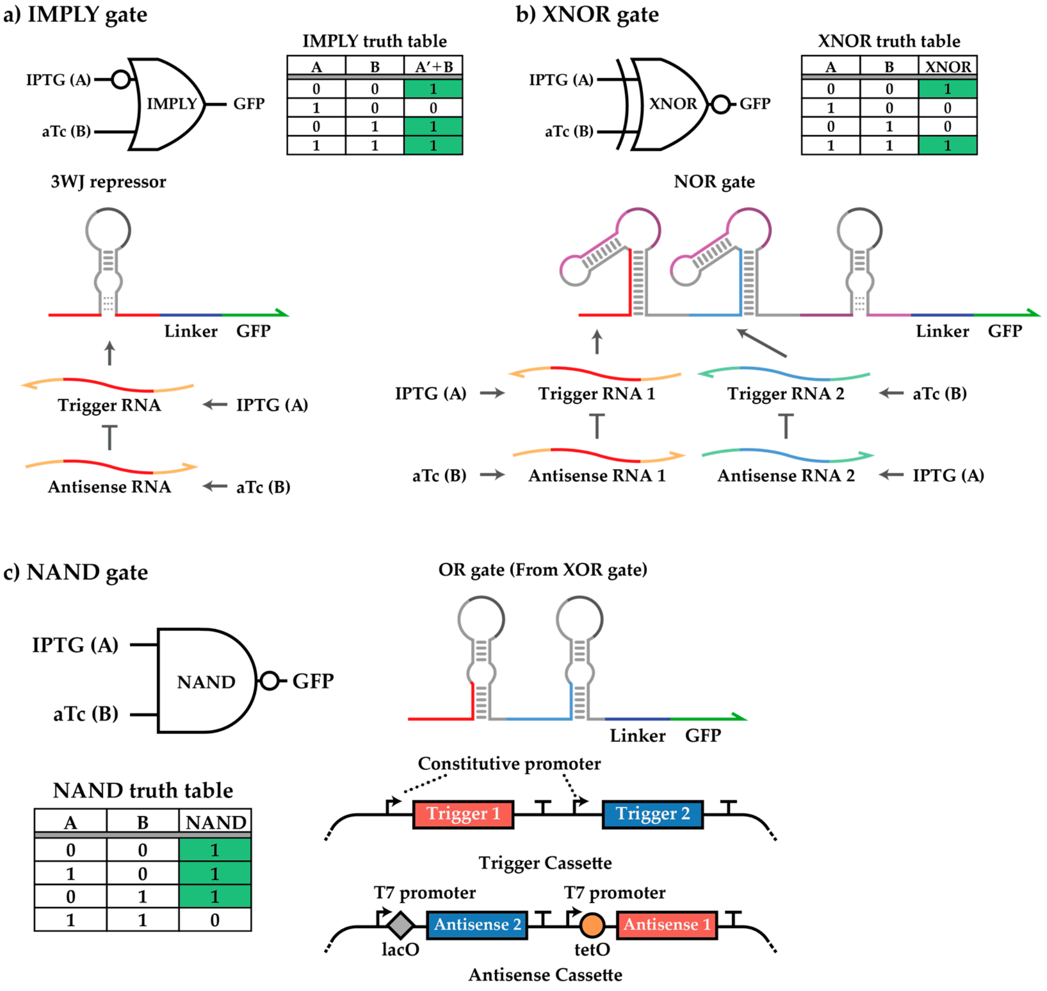

Supplementary Figure S17. Schematics of IMPLY, XNOR and NAND gates based on toehold switch and antisense RNAs. IMPLY gate and XNOR gate can be constructed using 3WJ repressor [1]. IMPLY gate has opposite truth table to NIMPLY gate. Equivalent to the operation of the NIMPLY gate, the IMPLY gate can also be made by arranging trigger RNA and antisense RNA inducers differently over 3WJ repressor. In addition, an XNOR gate can be easily made by using a NOR gate instead of the OR gate used in the XOR gate. On the other hand, NAND gates are distinguished from XOR gates in the (0,0) input state. Therefore, a NAND gate can be built by simply changing the T7 promoter of both triggers to a constitutive promoter.

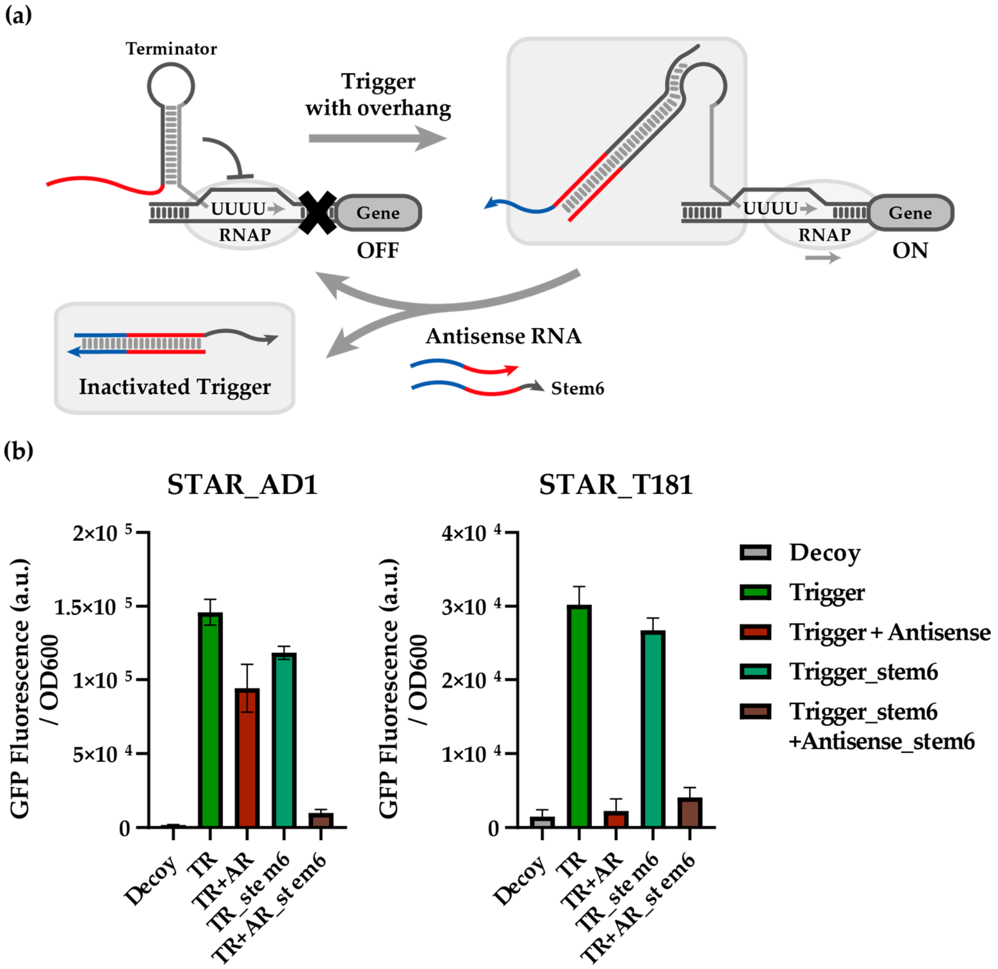

Supplementary Figure S18. Repressibility of antisense RNA in small transcription activating RNA (STAR) [2]. Antisense RNA was initially designed to detach trigger RNA from STAR switch only in the toehold region. In the case, weak repressibility was detected on STAR AD1. Otherwise, all antisense RNA can properly repress the trigger RNA activity on STAR AD1 and STAR T181. T7 RNA polymerase was induced by 1 mM IPTG in *E. coli* BL21 DE3 strain. GFP fluorescence was measured on the microplate reader (error bars indicate ±SD from three biological replicates).

References

1. Kim, J.; Zhou, Y.; Carlson, P. D.; Teichmann, M.; Chaudhary, S.; Simmel, F. C.; Silver, P. A.; Collins, J. J.; Lucks, J. B.; Yin, P.; Green, A. A., De novo-designed translation-repressing riboregulators for multi-input cellular logic. *Nat Chem Biol* **2019,** 15, (12), 1173-1182.
2. Chappell, J.; Takahashi, M. K.; Lucks, J. B., Creating small transcription activating RNAs. *Nat Chem Biol* **2015,** 11, (3), 214-20.
3. Green, A. A.; Kim, J.; Ma, D.; Silver, P. A.; Collins, J. J.; Yin, P., Complex cellular logic computation using ribocomputing devices. *Nature* **2017,** 548, (7665), 117-121.
